# Comparative Genomic Analysis Reveals Genetic Variation and Adaptive Evolution in the Pathogenicity-Related Genes of *Phytophthora capsici*

**DOI:** 10.3389/fmicb.2021.694136

**Published:** 2021-08-13

**Authors:** Joung-Ho Lee, Muhammad Irfan Siddique, Jin-Kyung Kwon, Byoung-Cheorl Kang

**Affiliations:** Department of Agriculture, Forestry and Bioresources, College of Agriculture and Life Sciences, Research Institute of Agriculture and Life Sciences, Plant Genomics and Breeding Institute, Seoul National University, Seoul, South Korea

**Keywords:** *de novo* hybrid assembly, comparative genomic analysis, pathogenicity-related genes, gene family expansion, variant calling

## Abstract

*Phytophthora capsici* is an oomycete pathogen responsible for damping off, root rot, fruit rot, and foliar blight in popular vegetable and legume crops. The existence of distinct aggressiveness levels and physiological races among the *P. capsici* population is a major constraint to developing resistant varieties of host crops. In the present study, we compared the genomes of three *P. capsici* isolates with different aggressiveness levels to reveal their genomic differences. We obtained genome sequences using short-read and long-read technologies, which yielded an average genome size of 76 Mbp comprising 514 contigs and 15,076 predicted genes. A comparative genomic analysis uncovered the signatures of accelerated evolution, gene family expansions in the pathogenicity-related genes among the three isolates. Resequencing two additional *P. capsici* isolates enabled the identification of average 1,023,437 SNPs, revealing the frequent accumulation of non-synonymous substitutions in pathogenicity-related gene families. Furthermore, pathogenicity-related gene families, cytoplasmic effectors and ATP binding cassette (ABC) transporters, showed expansion signals in the more aggressive isolates, with a greater number of non-synonymous SNPs. This genomic information explains the plasticity, difference in aggressiveness levels, and genome structural variation among the *P. capsici* isolates, providing insight into the genomic features related to the evolution and pathogenicity of this oomycete pathogen.

## Introduction

*Phytophthora capsici* is a major limiting factor in the production of several economically important cultivated species, causing losses in plants grown in both covered and open-field conditions (Foster and Hausbeck, 2010). Soil fumigation with fungicides is ineffective at controlling this pathogen (Rocha, 2014), which highlights the importance of building disease resistance into breeding programs.

The diverse geographical range of *P. capsici* isolates greatly affects the development of resistant varieties of crop plants. Earlier studies reported differences in the virulence and aggressiveness levels of *P. capsici* isolates targeting vegetable hosts (Foster and Hausbeck, 2010). Host-dependent aggressiveness has also been observed in this pathogen; for example, no substantial aggressiveness differences were observed among isolates when inoculated into cucumber (*Cucumis sativus*), while the same isolates showed varied aggressiveness levels when inoculated into tomato (*Solanum lycopersicum*) and pepper (*Capsicum annuum*) (Granke et al., 2012). Furthermore, 24 *P. capsici* isolates were grouped into 6 different groups based on aggressiveness when inoculated into pumpkin (*Cucurbita moschata*) seedlings, each corresponding to random amplified polymorphic DNA marker groups (Islam et al., 2005). Moreover, the existence of several races of *P. capsici* in different geographical regions infecting different plant organ is a major challenge to breed resistant cultivars. At least 45 physiological races of *P. capsici* have been reported to cause root rot and foliar blight in pepper (Barchenger et al., 2018). The high incidence of genetic diversity identified in *P. capsici* has been ascribed to sexual reproduction, mutation, and a loss of heterozygosity (Lamour et al., 2012; Jiang et al., 2015; Barchenger et al., 2018); however, no genomic studies have yet explored these variations in *P. capsici* isolates.

To determine the biological processes involved in host adaptation, a laboratory-bred inbred isolate, LT1534, was sequenced as a *P. capsici* reference genome using Sanger sequencing-based technology (Lamour et al., 2012). Genomic studies revealed that pathogenicity-related genes in *Phytophthora* spp. are commonly located in gene-poor regions dispersed within repetitive regions (Raffaele and Kamoun, 2012; Cui et al., 2019). Obtaining a more highly accurate genome sequence is crucial for detecting the prime genomic regions related to pathogenicity; for example, by leveraging the Pacific Biosciences (PacBio; Menlo Park, CA, United States) platform, researchers yielded a whole-genome assembly of *P. cactorum* that sized 121.5 Mb, almost 46% of which comprised repetitive sequences (Yang et al., 2018). Analogously, the haplotype-based assembly of *P. ramorum* generated using PacBio long-reads was used to identify more repeats (54%) than were detected in the previously assembled genome (29%), in addition to the identification of new effector-coding genes (Tyler et al., 2006; Mathu Malar et al., 2019). Recently, the *P. capsici* genome was reassembled by two research groups; one team used the HiSeq X Ten (Illumina, San Diego, CA, United States) platform to sequence six isolates, generating an average assembly size of 53.4 Mb (Reyes-Tena et al., 2019), whereas the other researchers used the Oxford Nanopore Technologies (Oxford, United Kingdom) MinION platform, which yielded a 95.2 Mb assembly (Cui et al., 2019). Nevertheless, sequencing multiple *P. capsici* isolates for a better comparative genomic analysis has not previously been reported.

Previous studies have suggested that the *Phytophthora* species have undergone several widespread genome duplications, resulting in a diverse repertoire of transposable elements, increased gene contents, and larger genome sizes (Jiang et al., 2005; Haas et al., 2009). Oomycete pathogens contain an extensive and divergent repertoire of expanded gene families, most of which encode proteins directly or indirectly involved in pathogenicity, such as glycoside hydrolases or NEP1-like proteins (Seidl et al., 2012). The RxLR and crinkler (CRN) proteins involved in pathogenicity are also encoded by expanded gene families in oomycete pathogens (Haas et al., 2009; Seidl et al., 2012). A recent study also suggested that the pathogenicity of *P. cactorum* might be related to the expansion and positive selection of gene families following a whole-genome duplication, along with gene losses (Yang et al., 2018); however, a comparative genomic analysis has not been performed for *P. capsici* isolates with diverse aggressiveness levels.

Despite the availability of the *P. capsici* reference genome, a comprehensive and thorough analysis of the similarity of the various *P. capsici* isolates is still lacking. The objective of the present study was to compare the genomes of five *P. capsici* isolates exhibiting different aggressiveness profiles to reveal their variation at the genomic level. The *P. capsici* isolates were categorized *in vitro* and *in planta* for their aggressiveness levels. The *de novo* genomes of three *P. capsici* isolates were generated using a hybrid assembly combining short-read (Illumina) and long-read (PacBio) data. A comprehensive genomic analysis was then performed using the *de novo* assembled contigs and the resequencing data of two additional *P. capsici* isolates to elucidate the genetic and evolutionary mechanisms underlying the differences in *P. capsici* aggressiveness levels.

## Materials and Methods

### Pathogen Isolates and DNA Extraction

The *P. capsici* isolates MY-1, JHAI1–7, KPC-7, Pc038, and PEP (Supplementary Table 1) used in the current study were kindly provided by KRICT (Korea Research Institute of Chemical and Technology), Daejeon, Korea; Kyungpook National University, Daegu, Korea; and East–West Seed Thailand, Nonthaburi, Thailand (Supplementary Table 1). The MY-1, JHAI1–7, and KPC-7 isolates were reported to have low, medium, and high aggressiveness levels, respectively (Jo et al., 2014; Liu et al., 2014; Siddique et al., 2019), while Pc038 (Abebe et al., 2016) and PEP (unpublished data) were both highly aggressive. The *P. capsici* isolates were re-isolated from diseased plant tissues before being used for aggressiveness level differentiation. For the high-quality DNA extraction, the *P. capsici* isolates were cultured in V8 agar medium.

For DNA extraction, small round blocks (1 cm) of mycelium tip harvested with a cork borer from actively growing cultures were used to inoculate 250 mL Erlenmeyer flasks containing 200 mL autoclaved potato dextrose broth. The cultures were incubated at 27 ± 2°C. After 5–10 days, the mycelia were harvested by filtration through cheesecloth, blotted dry with sterile paper towels, and stored at −80°C. High-molecular-weight DNA was isolated and purified using a modified fungal CTAB method, as described in detail in a previous study (Zelaya-Molina et al., 2011). The purity and quality of the isolated DNA samples were evaluated using electrophoresis on a 0.8% agarose gel and with a NanoDrop 2000 Spectrophotometer (Thermo Fisher Scientific, Waltham, MA, United States).

### Growth and Aggressiveness Evaluation in the Laboratory and the Greenhouse

The three *P. capsici* isolates (KPC-7, JHAI1–7, and MY-1) were subjected to growth and aggressiveness evaluation in the laboratory and greenhouse. The inoculum was prepared and adjusted as described in a previous study (Siddique et al., 2019). A set of 20 plants for susceptible pepper (*C. annuum*) cultivar “Tean” was inoculated with each of three isolates (KPC-7, JHAI1–7, and MY-1) as described previously (Siddique et al., 2019). The disease responses were observed as the symptoms that appeared on the stem collars.

In addition, a foliage aggressiveness test was performed using a detached leaf assay using three *P. capsici* isolates (KPC-7, JHAI1–7, and MY-1). Leaves of the susceptible cultivar “Tean” were harvested at the mature stage, placed on sterile moist filter papers inside Petri dishes to maintain the ambient humidity, and incubated at 27 ± 2°C. The inoculum density was adjusted to 10,000 spores/mL, and 20 μL of inoculum was dispensed onto the center of each leaf. Sterile distilled water was used as a mock control. The infection severity was assessed as the lesion size on the leaf surface at 3 and 6 days post inoculation (dpi).

The mycelium growth rates of the three *P. capsici* isolates (KPC-7, JHAI1–7, and MY-1) were determined for two different growth mediums under the same temperature and humidity at room temperature (27 ± 2°C). Small (1-cm) round blocks of actively growing mycelium were placed on the center of Petri plates filled with solid media comprising V8 agar or potato dextrose agar (PDA). The experiment was conducted with three replications per treatment. The colony diameters were measured every 24 h until the 90 mm plates were fully covered with mycelia.

### Whole Genome Sequencing and *de novo* Assembly

The genome sequencing of the three *P. capsici* isolates with different aggressiveness levels (MY-1, JHAI1–7, and KPC-7) was performed using both an Illumina HiSeq 4000 system and a PacBio Sequel system. To estimate the genome size before assembly, Jellyfish version 2.2.10 (Marçais and Kingsford, 2011) was used to count the frequency of *k*-mers with a *k*-mer size of 21. The genome size was estimated by the total number of nucleotides per peak depth of the *k*-mer frequency distribution. GenomeScope version 2.0 (Ranallo-Benavidez et al., 2020) was used to estimate the heterozygosity and size of the genomes. Assemblies with a 50× coverage of 101-bp reads were generated using Illumina paired-end sequencing (Supplementary Table 2). Additionally, 100× Sequel read was obtained with an average length of 9 kb, which is sufficient to achieve a *de novo* assembly. For the hybrid assembly, the MaSuRCA genome assembly package version 3.2.9 (Zimin et al., 2013) was used with the default parameters. After completing the genome assembly, HaploMerger2 (Huang et al., 2017) was used to compute the haploid state from the diploid assembled genome. The assembled genomes were validated for accuracy and completeness using BUSCO version 3.1.0 with the eukaryota dataset (Waterhouse et al., 2018). The reference *P. capsici* genome (LT1534) (Lamour et al., 2012) was compared with the newly assembled genomes to identify their collinearity using the nucmer (–mincluster = 1000) module in the MUMMER4 package (Marçais et al., 2018).

### Resequencing Analysis of *P. capsici*

Two isolates, Pc038 from Korea and PEP from Thailand, were additionally sequenced for a better comparative analysis using the Illumina HiSeq 4000 system only. The reads were aligned to the published *P. capsici* genome (LT1534) using Burrows-Wheeler Aligner version 0.7.17 (Li and Durbin, 2009). The alignment file was processed using Picard tools version 2.18.16 to sort the alignment information, remove duplicates, and group reads. The variant calling of all isolates was performed using GATK HaplotypeCaller version 3.8, and SNPs were filtered with several options (DP < 10; QUAL < 30; QD < 5.0). Variants were annotated using snpEff version 4.11 (Cingolani et al., 2012) to annotate the SNPs with a gene function. AgriGO version 2.0 and REVIGO were used for the gene ontology (GO) term enrichment analysis [hypergeometric statistical test, FDR (Hochberg) for multi-test adjustment, generic GO slim options] (Supek et al., 2011; Tian et al., 2017). The functions of the variant-containing annotated genes were predicted using BLAST2GO version 5 (Götz et al., 2008). All SNP calling sets of five *P. capsici* isolates are provided in Supplementary Data 1.

### Orthology Analysis and Structural Gene Annotation

Gene orthology analyses were performed using OrthoFinder version 2.3.5 (Emms and Kelly, 2015) following the default options for *P. capsici* isolates, including the previously reported reference genome (LT1534), and a Venn diagram was plotted using custom R scripts (R version 3.5.0). The LT1534 reference genome was reannotated using the same methods to compensate the effect of different annotation pipelines. For the precise structural annotation of genes, a custom *P. capsici* repeat library was constructed using RepeatModeler version 1.0.11^1^ by combining the oomycete repeat sequence library with the three *de novo* repeat libraries for the assembled genomes. This repeat sequence library was then incorporated into the masked repeat sequences using RepeatMasker version 1.332^2^ (Vetukuri et al., 2018) before being used for the gene prediction.

Finally, the structural genome annotation was performed in two rounds using MAKER 2.31.10 (Holt and Yandell, 2011). First, evidence-driven gene prediction was performed by importing the expressed sequence tag sequences of *P. capsici* (Lamour et al., 2012) and the peptide sequences of eight *Phytophthora* species (*P. capsici*, *P. infestans*, *P. kernoviae*, *P. lateralis*, *P. parasitica*, *P. ramorum*, and *P. sojae*) and one closely related oomycete (*Hyaloperonospora arabidopsidis*) into the MAKER2 pipeline. The annotated files from the MAKER2 pipelines were trained into Augustus version 3.3.3 (Stanke and Morgenstern, 2005). Finally, Augustus, SNAP (Korf, 2004), GeneMark-ES version 3.6.0 (Ter-Hovhannisyan et al., 2008), and Exonerate version 2.2.0 (Slater and Birney, 2005) were used for the prediction of genes in the *de novo* assembled genomes.

### Functional Annotation and Classification

Peptide sequences from the structural annotation were used for the functional annotation of the genes. InterProScan version 5.34–73.0 (Quevillon et al., 2005) was used to analyze the gene function and to obtain functional structures and GO terms. Genes encoding carbohydrate-active enzymes (CAZymes) were predicted by scanning the peptide sequences using HMMER 3.0, DIAMOND, and Hotpep in the online tool dbCAN2 (Zhang et al., 2018). The genes encoding CAZymes were selected for further analysis after their confirmation by two programs. To further classify the annotated proteins, the annotated genes were mapped against the protein classification database. The protease families were further predicted and classified according to the criteria in the MEROPS peptidase database (Rawlings et al., 2018). Peroxidase families were further analyzed by BLAST-searching against the peroxidase protein database in the UniProt database.^3^ Transporter proteins were additionally confirmed by searching the Transporter Classification Database (Saier et al., 2006) using BLAST. Pathogenicity-associated genes were putatively identified by searching the pathogen-host interaction database (PHI) (Urban et al., 2020) using BLAST. The *e*-value cut-off for all BLAST searches was set to 1E–5. Secondary metabolite-related genes were annotated using the antiSMASH version 5.1.0 pipeline and the SMURF web-based tool (Khaldi et al., 2010; Blin et al., 2019).

The R package “effectR” (Tabima, 2019) was used to annotate the effector proteins in the genome. Briefly, amino acids were predicted using the “getorf” function in EMBOSS, and were then merged with the predicted gene sequences. Finally, the obtained protein models were used to predict the effector proteins using a combination of regular expression statements (RxLR or LFLAK) and hidden Markov model-based approaches using the effectR package. The RxLR effectors were classified with an effectR annotation if signaling evidence was obtained from SignalP5.0 and TargetP but there was no evidence of a transmembrane motif in the TMHMM version 2.0 results (Krogh et al., 2001; Almagro Armenteros et al., 2019; Armenteros et al., 2019). The CRN effectors were annotated based on the effectR prediction results in the absence of transmembrane motif evidence from TMHMM2.0. All structural and functional annotated gene sets of three *P. capsici* isolates are provided in Supplementary Data 2.

### Evolutionary Analysis and Positively Selected Genes

The protein sequences of closely related pathogens, such as *P. tricornutum*, *Thalassiosira pseudonana*, *Saprolegnia parasitica*, *P. sojae*, *P. infestans*, *P. kernoviae*, *P. lateralis*, *P. parasitica*, *P. ramorum*, *H. arabidopsidis*, *Pythium irregulare*, and *Albugo laibachii*, as well as two outgroup species (*S. parasitica* and *T. pseudonana*), were used to calculate the divergence time of the *P. capsici* isolates (Sun et al., 2017). Single-copy orthologs were used to generate the phylogenetic tree using RAxMLHPC-PTHREADS-AVX (Stamatakis, 2014) with 1000 bootstrap replicates (-m PROTGAMMADAYHOFF; -p 12345; -x 12345; -# 1000). The phylogenetic trees were analyzed using codeml and MCMCTree in the PAML4.9i package (Yang, 2007) to estimate the divergence time.

To observe the evolutionary relationship of the gene families, the orthologs were analyzed using CAFE version 4.2.1 (De Bie et al., 2006). To avoid an erroneous gene family evolutionary signal from the gene annotation pipeline, the “cafeerror” function was also used in CAFE to correct for genome assembly errors. Only significantly expanded or contracted gene families identified using CAFE (*p*-value < 0.01) were used in further analyses. The positive selection signals were estimated using codeml in the PAML package (Jeffares et al., 2015). LRT statistics were used to observe the positive selection signals in the codeml output.

## Results

### Aggressiveness Evaluation and Characterization of *P. capsici* Isolates

**FIGURE 1:**
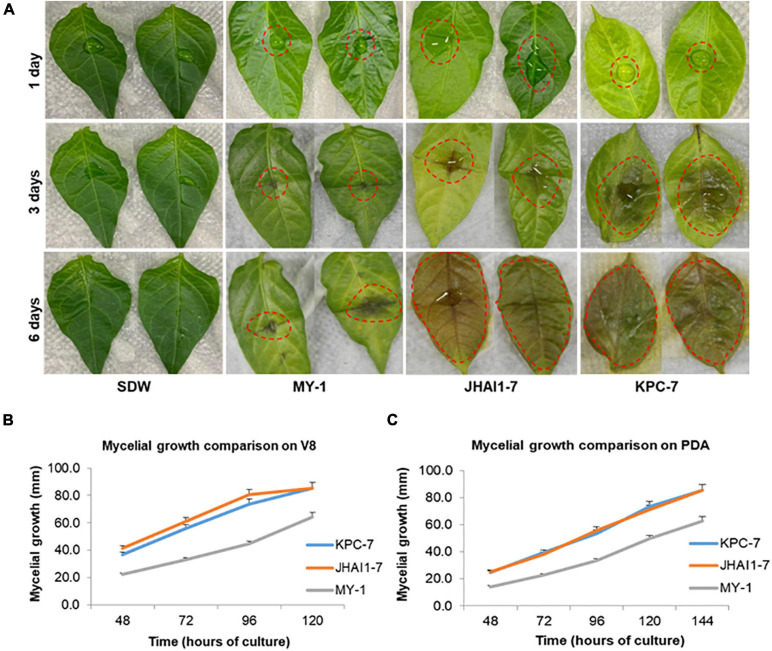
Detached leaf assay and mycelium growth comparison for aggressiveness assessment. **(A)** Detached leaf assay. Sterile distilled water (SDW) was used as a mock control. The red dotted lines indicate the *P. capsici*-infected lesion area. **(B)** Mycelial growth on V-8 agar medium. **(C)** Mycelial growth on potato dextrose agar (PDA) medium. The bars indicate the standard error of means *n* = 3.

Three *P. capsici* isolates, KPC-7, JHAI1–7, and MY-1 (Supplementary Table 1), showed clear differences in their symptom development and aggressiveness levels on susceptible pepper plants. Disease symptoms were first noticed at 3, 5, and 10 dpi for KPC-7, JHAI1–7, and MY-1, respectively (Supplementary Figure 1). The inoculated plants had completely wilted and died at 10 and 14 dpi for KPC-7 and JHAI1–7, respectively; however, the susceptible plants did not fully wilt within 3 weeks of inoculation with MY-1 (Supplementary Figure 1). In addition, aggressiveness was also confirmed using a detached leaf assay. The leaves inoculated with isolate MY-1 showed the least yellowing and hygrophanous lesion formation at 3 and 6 dpi (Figure 1A). The isolate JHAI1–7 produced an intermediate level of infection on the leaf surface at 3 dpi, while the whole leaf surface was covered with mycelial infection at 6 dpi. KPC-7 showed high levels of aggressiveness and caused lesions across the entire leaf at 3 dpi. These results confirmed the low, medium, and high aggressiveness levels of the MY-1, JHAI1–7, and KPC-7 isolates, respectively, *in planta*.

The growth rates of the isolates were compared on two different nutrient media (V8 agar and PDA). The isolates KPC-7, JHAI1–7, and MY-1 grew to an average colony size of 36.7, 41.3, and 22.2 mm, respectively, on the V8 agar medium after 48 h of culture (Figure 1B and Supplementary Figures 2A–C). The KPC-7 and JHAI1–7 mycelia had spread throughout the Petri dishes (90 mm diameter) after 5 days of culture, while the MY-1 colonies had an average diameter of 64.3 mm (Figure 1B). The average KPC-7 and JHAI1–7 colony sizes on PDA were 24.7 and 25.3 mm, respectively, after 48 h of culture, whereas the MY-1 colonies were 14.2 mm in diameter (Figure 1C and Supplementary Figures 2D–F). The KPC-7 and JHAI1–7 mycelia had spread throughout the Petri dishes at 6 days after culturing, while a 63-mm average colony diameter was recorded for MY-1 (Figure 1C). These results indicated a correlation of isolate the aggressiveness level with the mycelium growth rate on growth media, with highly aggressive isolate showing a more rapid mycelial growth.

### Overview of the *de novo* Hybrid Genome Assembly

Assemblies with a 50× coverage of 101-bp reads were generated using Illumina paired-end sequencing (Supplementary Table 2). Complementary to this, 100× PacBio Sequel reads were obtained with an average length of 7.2 kb, which was sufficient to accomplish a *de novo* assembly (Supplementary Table 2). A genome *k*-mer analysis revealed that the estimated genome sizes of the three isolates ranged from 69.89 to 76.90 Mb (Supplementary Figure 3). In the *k*-mer distribution plots, two peaks with similar frequencies represented the diploid stage of the three *P. capsici* isolates, with a high heterozygosity in each genome (Supplementary Figure 3). The *de novo* hybrid genome assembly generated 74.9, 76.8, and 76.6 Mb assemblies for KPC-7, JHAI1–7, and MY-1, respectively (Table 1), which was consistent with the estimated genome sizes. The genome assembly process resulted in 521, 472, and 549 scaffolds for KPC-7, JHAI1–7, and MY-1, respectively, with GC contents of 50.9, 51.0, and 50.9% and N50 values of 6.63, 7.98, and 6.35 Mb, respectively. The longest and shortest scaffold lengths in the assembled genomes were 2.7 Mb and 657 bp for KPC-7, 2.5 Mb and 1,229 bp for JHAI1–7, and 2.7 Mb and 723 bp for MY-1. The KPC-7, JHAI1–7, and MY-1 genomes were predicted to contain 15,322, 16,457, and 16,343 genes, respectively. Similar gene density patterns were observed in all three isolates, and the average log_2_ value of the gene densities was 9.074 (Supplementary Figure 4). A total of 1,389, 1,350, and 1,256 tRNAs were identified in the KPC-7, JHAI1–7, and MY-1 genomes, respectively. Furthermore, 2,521 small nucleolar RNAs (snoRNAs) were predicted in KPC-7, 2,663 in JHAI1–7, and 2,526 in MY-1 (Table 1). An average 37.46 Mb (49.19%) of the three isolate genomes was predicted to contain repetitive elements. The major repetitive elements were LTR/Gypsy elements, which accounted for 20% of the three genome sequences (Supplementary Table 3). A BUSCO completeness analysis revealed a greater than 90% accuracy for the assembled genomes (Table 1). The accuracy of the genome assemblies were further evaluated by comparing them with the previously published *P. capsici* reference genome (Lamour et al., 2012). The dot plot analysis showed the syntenic relationship between the genome assemblies and the reference genome, LT1534 (Supplementary Figure 5).

**TABLE 1:** Statistics of the *P. capsici* genome assembly result.

	**MY-1**	**JHA1–7**	**KPC-7**	**LT1534 v11.0**
Number of scaffolds	521	472	549	917
GC content (%)	50.96	51.01	50.91	44.14
Total size (bp)	76,624,583	76,839,304	74,955,867	64,023,748
Longest scaffold (bp)	2,720,928	2,514,355	2,715,842	2,170,955
Min scaffold length (bp)	723	1,229	657	1,001
N50 of scaffolds (bp)	635,553	798,104	663,153	705,730
Number of genes	16,343	16,457	15,322	19,805
Number of tRNAs	1,256	1,350	1,389	–
Number of snoRNAs	2,526	2,663	2,521	–
Percentage of repeats (%)	49.49	49.34	48.76	–
Repeat sequence length (bp)	37,919,069	37,915,333	36,550,822	–
BUSCO completeness (%)	92.10	92.10	92.40	–
Remarks	This study	This study	This study	Lamour et al., 2012

### Gene Orthology Analysis

**FIGURE 2:**
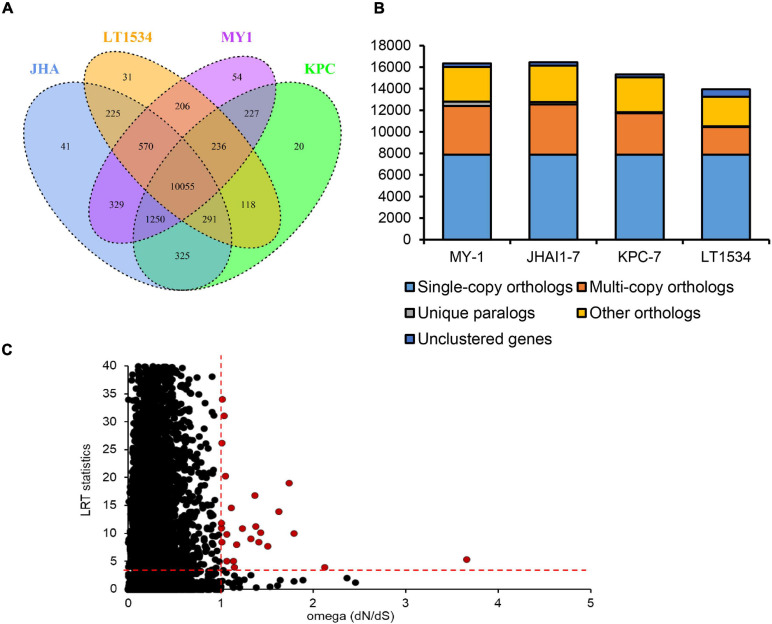
Gene orthology analysis of the *P. capsici* genome assembly. **(A)** Venn diagram showing the numbers of unique and shared gene families among four *P. capsici* isolates. **(B)** Distribution of orthologous genes in the four *P. capsici* strains. **(C)** Positive selection signals of the single-copy orthologous genes. The red circles indicate positively selected single-copy orthologous genes, while the black circles represent orthologous gene pairs that were not significantly positively selected. The red dotted lines represented the criteria for positive selection (dN/dS > 1.0; Chi-square value for LRT statistics ≥ 3.8416).

The clustering of predicted proteins from the three *P. capsici* isolates in this study and the previously sequenced (Lamour et al., 2012) isolate LT1534 revealed 13,978 orthologous groups containing 60,453 proteins (Figure 2). Several unique protein clusters specific to each isolate were identified in orthology analysis (Figure 2A). In detail, the previously sequenced isolate LT1534 contained 31 unique gene clusters, whereas the KPC-7, JHAI1–7, and MY-1 isolates were shown to have 20, 41, and 54 gene clusters, respectively (Figure 2A). A total of 10,055 orthologous groups were common among the three newly sequenced isolates, including 7,877 single-copy orthologous groups, whereas 3,852, 4,573, and 4,534 multiple-copy orthologs were detected in KPC-7, JHAI1–7, and MY-1, respectively (Figure 2B). In multi-copy orthologs, genes containing reverse transcriptase domain or retrotransposon gag domain were found to be expanded in new sequenced three isolates than reference *P. capsici* isolate, LT1534. Furthermore, 95 unique paralogs were detected in KPC-7, 205 in JHAI1–7, and 411 in MY-1 (Figure 2B). By contrast, fewer than 330 genes were not clustered. Positive selection, which can be observed as genetic variants or nucleotide diversity in coding sequences, is important evolutionary evidence of the aggressiveness and race differentiation in a given species of pathogen (Ye et al., 2016). To detect the evidence of positive selection in the three *P. capsici* isolates, as well as the reference genome of isolate LT1534, a total of 7,877 single-copy orthologs from the *P. capsici* proteomes were analyzed. A total of 26 gene orthologs comprising 104 genes showed a significantly positive selection signal (dN/dS > 1 and *p*-value < 0.05, LRT statistics, Chi-square test) (Figure 2C). Nine of them underwent strong positive selection (dN/dS > 1.2), including those encoding translation initiation factor 4G, a Pogo transposable element, an RxLR effector, the origin of replication complex, and an oxidoreductase (Figure 2C and Supplementary Table 4).

### Functional Annotation of Pathogenicity-Related Genes

#### Carbohydrate-Active Enzymes

**FIGURE 3:**
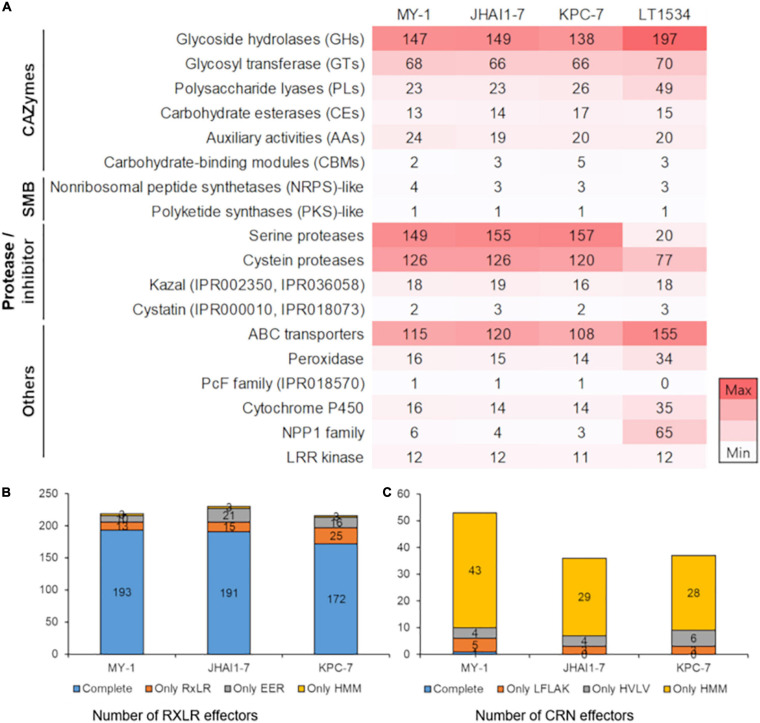
Composition of the pathogenicity-related protein families in four *P. capsici* isolates. **(A)** The number of annotated genes in four *P. capsici* isolates, including the reference genome. A darker color represents a higher number of genes in the protein families. CAZymes, carbohydrate-active enzymes; SMB, secondary metabolite biosynthesis genes. The number of annotated genes is represented by the color intensity from white (low) to red (high) color. **(B)** The number of RxLR effector genes in three *P. capsici* isolate genomes. Complete: both RxLR and EER sequence motifs were detected; Only RxLR: only RxLR sequence motifs were detected; Only EER: only EER sequence motifs were detected; Only HMM: no sequence motifs, but HMM profiles were detected. **(C)** The number of CRN effector genes in three *P. capsici* isolate genomes. Complete: both LFLAK and HVLV sequence motifs were detected; Only LFLAK: only LFLAK sequence motifs were detected; Only HVLV: only HVLV sequence motifs were detected; Only HMM: no sequence motifs, but HMM profiles were detected.

The CAZymes play a direct role in pathogenicity and virulence by instigating plant cell wall degradation (Lyu et al., 2015). A total of 272, 274, and 277 transcripts encoding CAZymes were detected in the assembled genomes of the KPC-7, JHAI1–7, and MY-1 isolates, respectively (Figure 3). These secreted CAZymes include glycosyl hydrolases (GHs), glycosyl transferases (GTs), polysaccharide lyases (PLs), auxiliary activities (AAs), carbohydrate-binding modules (CBMs), carbohydrate esterases (CEs), and signal peptide families. The isolate MY-1 contained 147 GHs, 68 GTs, 23 PLs, 24 AAs, 2 CBMs, and 13 CEs, while JHAI1–7 contained 149 GHs, 66 GTs, 23 PLs, 19 AAs, 5 CBMs, and 14 CEs. The isolate KPC-7 contained 138 GHs, 66 GTs, 26 PLs, 20 AAs, 5 CBMs, and 17 CEs. The total numbers of CAZymes identified in these three isolates was lower than that in the reference genome (351 CAZymes in LT1534), but the number of secreted CAZymes was comparable in all isolate genomes: 113 in KPC-7, 126 in JHAI1–7, 112 in MY-1, and 103 in previously sequenced isolate LT1534 (Supplementary Figure 6).

#### Secondary Metabolite Biosynthesis-Related Genes

Some genes encoding known virulence-associated proteins were further characterized for the three *P. capsici* isolates. Four secondary metabolite biosynthesis (SMB) genes were identified in KPC-7 and JHAI1–7, with five detected in MY-1. Likewise, five SMB-related genes were predicted in the previously sequenced LT1534 genome (Figure 3A). Three non-ribosomal peptide synthetase (NRPS)-like genes were identified in KPC-7 and JHAI1–7, whereas four such genes were detected in MY-1. One polyketide synthase (PKS)-like gene was identified in our three *P. capsici* isolates, which was consistent with the previously sequenced LT1534 genome.

#### Protease and Protease Inhibitors

Protease-related gene families were detected in the three *P. capsici* isolates, containing between 440 and 458 genes; however, 523 proteases were reported in the LT1534 reference genome (Figure 3A and Supplementary Data 2). The difference might be due to the protease and inhibitor database composition. A total of 120 cysteine protease genes were detected in KPC-7, while 126 were identified in JHAI1–7 and MY-1. A further analysis led to the identification of 157, 155, and 149 serine protease genes in KPC-7, JHAI1–7, and MY-1, respectively (Figure 3A).

#### ATP Binding Cassette Transporters and Peroxidase

Between 108 and 120 ATP binding cassette (ABC) transporter gene were identified in the three *P. capsici* isolates, whereas the LT1534 genome contained 155 ABC transporter genes (Figure 3). This difference in number might also be attributed to the different annotation pipelines used. Similarly, 34 peroxidase genes were reported in LT1534, but only 14, 15, and 16 were identified in KPC-7, JHAI1–7, and MY-1, respectively (Figure 3A).

#### PcF, Cytochrome P450, Necrosis Inducing Protein 1, and Leucine Rich Repeat Kinase Families

The three *P. capsici* isolates each contained one PcF genes (Figure 3A), whereas 14 cytochrome P450 genes were identified in KPC-7 and JHAI1–7 with 16 detected in MY-1. In contrast, 35 cytochrome P450 genes were reported in LT1534. Furthermore, the detection of the necrosis inducing protein 1 (NPP1) family-related genes in the three *P. capsici* isolates was considerably low as only one was detected in KPC-7 and two in JHAI1–7 and MY-1 than 65 in LT1534 (Figure 3A). This incongruity may also be attributed to the different annotation pipelines and prediction criteria used. A total of 11 leucine rich repeat (LRR) kinase genes were identified in KPC-7, whereas 12 were present in JHAI1–7 and MY-1, consistent with the same number (12) detected in the LT1534 reference genome (Figure 3A).

#### Cytoplasmic Effectors

Crinkler and RxLR effectors are considered to be major contributors to the pathogenicity and virulence of *Phytophthora* species. Due to their small size and irreproducibility in genome, we further annotated the cytoplasmic effector proteins using the R package, “effectR.” The functional annotation for the RxLR effectors in the assembled genomes revealed 584 RxLR genes in KPC-7, 736 in JHAI1–7, and 658 in MY-1, but the number of RxLR effectors with targeting sequences for plant cells was 216 in KPC-7, 230 in JHAI1–7 and 219 in MY-1 (Figure 3B and Supplementary Data 2). A total of 345, 317, and 400 CRN effector genes were identified in KPC-7, JHAI1–7, and MY-1 by functional annotation, but the number of CRN effectors with a targeting signal for plant cells was 53 in MY-1, 36 in JHAI1–7, and 37 in KPC-7 (Figure 3C and Supplementary Data 2). The RxLR and CRN effector genes were clustered in the gene-sparse regions of the genome, following similar trends in other closely related *Phytophthora* spp. (Supplementary Figures 4, 7).

### Phylogenetic Relationship of the *de novo* Assembled *P. capsici* Genomes

**FIGURE 4:**
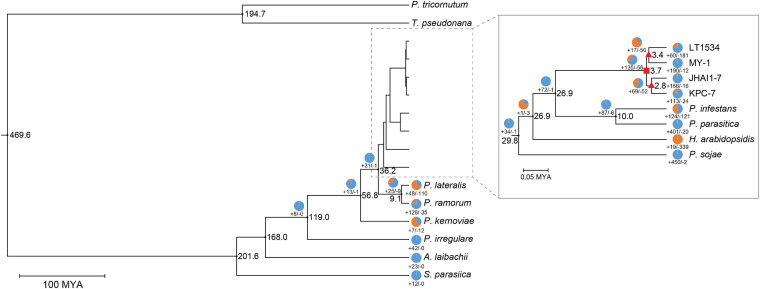
Phylogenetic relationships among oomycetes. The numbers at each node represent the estimated divergence time of the oomycete species from MCMCTree results. The circles and their associated numbers indicate the expansion/contraction in the number of significantly overrepresented gene families. Rectangle in nodes indicates the *P. capsici*-specific node, while triangles in nodes denotes the common ancestors of low (LT1534 and MY-1) and high (JHAI1-7 and KPC-7) aggressive isolates.

To determine the evolutionary relationships among the *P. capsici* isolates, a phylogenetic tree was constructed using 14 oomycete plant pathogens including two outgroup species (Figure 4). The topology of phylogenetic tree was similar to a previously reported tree (Sun et al., 2017), which strengthened the inferences made in our study. The timescale uploaded in the TimeTree database^4^ was used to estimate the divergence time of the three *de novo* assembled *P. capsici* isolates, as well as the previously sequenced isolate (LT1534) (Kumar et al., 2017). The estimated divergence time of the four *P. capsici* isolates was approximately 0.23 MYA (million years ago). The highly aggressive isolate, KPC-7, was more closely related to the moderately aggressive isolate, JHAI1–7, than the low-aggressive isolate, MY-1 (Figure 4). These results indicate that the high- (KPC-7) and medium-aggressive (JHAI1–7) isolates had a close evolutionary relationship, while the low-aggressive isolate is slightly more distantly related to them (Figure 4).

### Gene Family Expansion and Contraction

To identify the correlation between the aggressiveness level and the gene family expansion signals among the *P. capsici* isolates, we performed a comparative genomic analysis using the phylogenetic tree data in a gene family expansion and contraction analysis (Figure 4 and Supplementary Figure 8). A total of 135 gene families were observed to have significantly expanded and 56 contracted in the *P. capsici* clade compared with other *Phytophthora* species (rectangle node in Figure 4). The *P. capsici* phylogenetic node notably showed an expansion of the P-loop containing nucleoside triphosphate hydrolases (InterPro IPR027417), RxLRs (IPR031825), ankyrin repeat (IPR036770), and DNA helicase Pif1-like (IPR10285) gene families (Supplementary Table 5). In addition, the GO terms, GO:0004386 (helicase activity), GO:0017111 (nucleoside-triphosphatase activity), and GO:0016817 (hydrolase activity, acting on acid anhydrides), were significantly enriched in this *P. capsici* specific phylogenetic node (Figure 4 and Supplementary Table 6).

To confirm the aggressiveness-associated factors, the nodes of the common ancestors between the high-, medium- (KPC-7 and JHAI1–7) (Triangle node in Figure 4), and low-aggressive (MY-1) isolates were compared (Figure 4). We counted the number of InterPro (IPR) terms and performed GO term enrichment tests in two nodes. A total of 73 gene families, 17 expanded and 56 contracted, were identified in the low-aggressive node. Three InterPro terms, IPR012337 (ribonuclease H-like superfamily), IPR036291 [NAD(P)-binding domain superfamily], and IPR02347 (short-chain dehydrogenase/reductase), were detected in the contracted gene families, whereas the flavoprotein-related domains (IPR010089, IPR008254, and IPR029039) appeared in the expanded gene families at this node (Figure 4 and Supplementary Table 7). The GO terms, GO:0008289 (lipid binding), GO:0003676 (nucleic acid binding), and GO:0016788 (hydrolase activity, acting on ester bonds), were significantly enriched in the contracted gene families, while GO:0000166 (nucleotide binding) was enriched in the expanded gene families at this node (Supplementary Table 8).

A total of 121 gene families, 69 expanded and 52 contracted, were identified in the medium- and high-aggressive isolate node (Figure 4). Among these gene families, those associated with IPR001938 (thaumatin family), IPR037176 (osmotin/thaumatin-like superfamily), and IPR016024 (armadillo-type fold) were contracted, while the IPR terms IPR027417 (P-loop containing nucleoside triphosphate hydrolase), IPR010285 (DNA helicase Pif1-like), and IPR012337 (ribonuclease H-like superfamily) were associated with the expanded gene families in this node (Supplementary Table 7). Additionally, the RxLR effector domain (IPR03825) and protein kinase domain (IPR011009) were also detected in the expanded gene families in the medium- and high-aggressive isolates. Five GO terms, including GO:0006259 (DNA metabolic process), GO:0016817 (hydrolase activity, acting on anhydrides), and GO:0003677 (DNA binding), were enriched at this node. A total of 36 GO terms were significantly enriched in the expanded gene families in the high-aggressive node, including GO:0006259 (DNA metabolic process), GO:0006950 (response to stress), and GO:0050896 (response to stimulus) (Supplementary Table 8). Combining the results of the IPR term analysis with the GO term enrichment analysis revealed that the expanded gene families are frequently included the repertoire of rapidly evolving genes, such as effectors or transposable element domain-harboring proteins. These results indicated that rapidly evolving proteins not only affect the *P. capsici* clade, but also the rapid evolution of pathogenicity-related genes in the aggressive isolates.

### Whole Genome Comparison Based on Resequencing Analysis

To additionally identify the aggressiveness-associated factors in *P. capsici*, a total of five *P. capsici* isolates, including two additional highly aggressive isolates (Pc038 and PEP), were resequenced with an average 44.4× coverage using Illumina paired-end sequencing. After filtering, an average of 1,023,437 SNPs and 245,100 insertion/deletion mutations (InDels) were identified in the five isolate genomes (Supplementary Table 9). Among the SNPs, an average of 275,542 synonymous, 116,432 missense, 1,292 nonsense, and 605,663 intergenic SNPs were identified against the LT1534 reference genome. SNP densities were ranged from 5.37 to 8.11 and average nucleotide diversity was 0.0047. These results indicated the presence of significant variations in the genomes of the *P. capsici* isolates sequenced in our study in comparison with the previously sequenced reference genome of isolate LT1534. To validate the resequencing approach, we compared our SNP data with previously reported SNPs involved in mating type conversion (Lamour et al., 2012). Among five reported SNPs, two reported SNPs are correlated with the mating type of five *P. capsici* isolates, which is supporting that our resequencing data is sufficient for subsequent analysis (Supplementary Figure 9).

To identify the genes affected by the global variants, a GO term enrichment test was performed for the genes containing more than 20 disruptive variants (Ns > 20; variants for start/stop and missense codons) (Supplementary Table 10). A total of 75 GO terms in molecular function and one GO term in cellular components were enriched. The most enriched GO terms included ATPase activity (GO:0016887), nucleoside triphosphatase activity (GO:001711), hydrolase activity (GO:0016818, GO:0016817). In particular, GO terms, the nucleoside triphosphatase and hydrolase activity, are also enriched in the expanded gene families of highly aggressive isolates (Supplementary Table 8). Among 308 genes in enriched GO terms containing nucleoside triphosphatase activity (GO:0017111) (Supplementary Table 10), 152 ABC transporter encoding genes were included out of a total of 155 ABC transporter encoding genes in LT1534. These resequencing results also supported that ABC transporters are genes during *P. capsici* evolution like other virulence-associated effectors, RxLR or CRN.

### Evolutionary Footprints for the Pathogenicity-Related Genes in *P. capsici*

**FIGURE 5:**
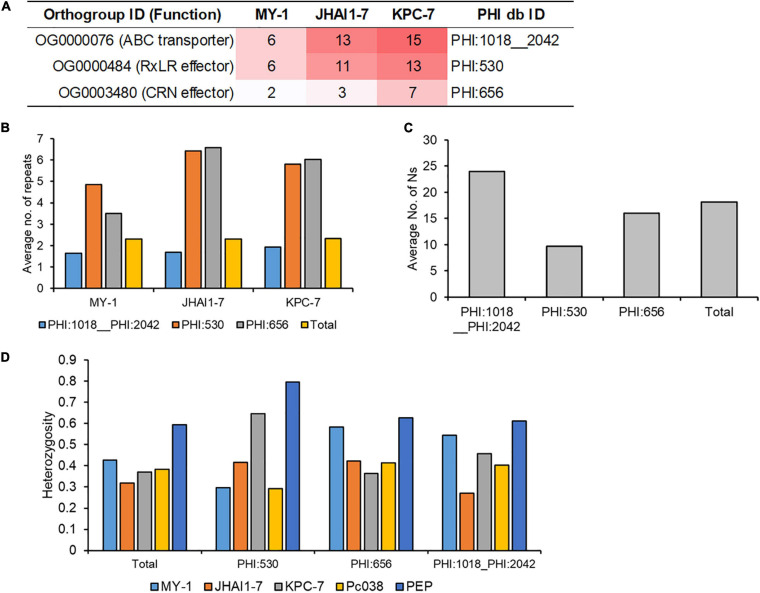
Genomic and reseqeuncing evidence for the evolution of the agressive isolates. **(A)** The number of protein families (corresponding to the expanded gene families) in three isolates. The number of protein families is represented by the color intensity from white (low) to red (high) color. Three protein families in plant-host interaction (PHI) database were represented by PHI ID (PHI:1018__2042: ABC3; PHI:530: Avr1b RxLR effector; crn1 CRN effector. **(B)** The number of repeat elements around 2.3 kbp (average distance of genes) in each protein family where total genes represent all annotated genes. **(C)** The number of disruptive SNPs in each gene family where total genes represent all annotated genes. **(D)** Heterozygosity levels in each gene family where total genes represent all annotated genes.

Combined with gene family expansion/contraction and resequencing results, we subsequently investigated three pathogenicity-associated gene families, ABC transporters, RxLR effectors, and CRN effectors (Figure 5). Three expanded gene families in high aggressive *P. capsici* isolates (ABC transporter: PHI:1018__PHI:2042; RxLR effector: PHI:530; CRN effector: PHI:656) showed significant differences in their numbers of constituent genes and expansion signals, which were associated with the aggressiveness levels of the isolates (Figure 5A).

Several mechanisms have been proposed to explain the adaptive evolution of phytopathogen into biotic/abiotic stress, such as two-speed genome theory or loss-of-heterozygosity (Lamour et al., 2012; Dong et al., 2015). To illustrate the evolutionary mechanisms of *P. capsici* adaptive evolution in five isolates, we investigated the distribution of repeat sequences in 5′- and 3′-region of gene sequences, disruptive nucleotides in exonic region, and heterozygosity level of the genic region to understand the evolutionary mechanism of these gene families, encoding CRN effectors, RxLR effectors, and ABC transporters. The average number of repeat elements in the CRN effector (PHI:656) and RxLR effector (PHI:530) genes were significantly higher than for all gene families (Figure 5B), whereas the number of repeat elements in the ABC transporter (PHI:1081_1042) was lower than that of all other gene families combined. The highest number of disruptive SNPs was detected in the ABC transporter (PHI:1081_1042) gene family (Figure 5C), but RxLR (PHI:530) and CRN effectors (PHI:656) have a lower average number of disruptive SNPs than that of total genes. Heterozygosity levels of resequenced strains were frequently distributed from 0.3 to 0.4 with the exception of a high level of heterozygosity in PEP isolate (Figure 5D). Even some RxLR effectors (PHI:530) in high aggressive isolates showed higher heterozygosity levels than the total gene family (Figure 5D), but significant trends of heterozygosity levels were not detected in three gene families. Representative SNP data of three gene families (PoAvh103 for RxLR; PcCRN4 for CRN; 6938 for ABC transporter) showed different and distinct heterozygosity patterns in five isolates (Supplementary Figure 10), which supported that the extensive mating and crossover induce the adaptive evolution of *P. capsici* isolates. For example, SNP patterns in PcAvh103 showed the patterns of MY-1 and that of PEP are similar, but different SNP patterns were observed in PcCRN4 and transcript 6938 (Supplementary Figure 10 and Supplementary Data 2). These results indicated that diverse evolutionary mechanisms, various mating and crossover between *P. capsici* isolates influence the evolution of the pathogenicity-related genes, such as RxLR effectors, CRN effectors, and ABC transporters.

## Discussion

Different levels of aggressiveness among *P. capsici* isolates have been observed depending on the host and geographical variation (Granke et al., 2012; Nawaz et al., 2018; Siddique et al., 2019), however, the genomic comparison has not been performed. The broad distribution of *P. capsici* isolates with diverse aggressiveness levels impacts the breeding and deployment of resistant varieties of host crops (Foster and Hausbeck, 2010). Previously, the virulence and aggressiveness of 126 *P. capsici* isolates from 12 countries was assessed for six different hosts, revealing a noticeable variation in virulence and aggressiveness on the same and diverse host (Granke et al., 2012). Another study reported virulence and aggressiveness variations among 17 *P. capsici* isolates when characterized using pepper as a host (Nawaz et al., 2018). Our characterization study, based on root inoculation and a detached leaf assay, revealed the striking variation in the aggressiveness levels of three *P. capsici* isolates. The symptom development on the host, growth pace of the colony formation on different growth media (V8 agar and PDA), and hygrophanous lesion areas on inoculated detached leaves demonstrated the difference in aggressiveness levels of the *P. capsici* isolates. Based on these observations, we categorized MY-1 as less aggressive, whereas JHAI1–7 and KPC-7 were designated as moderately and highly aggressive isolates, respectively.

The sequencing of the hemibiotrophic oomycetes and various *Phytophthora* spp. has opened up new avenues for the study of comparative genomics to explore the pathogenicity-related factors in these organisms (Jiang and Tyler, 2012; Lamour et al., 2012). Recent progress in long-read sequencing technologies has led to a significant improvement in genome assemblies, particularly due to the amalgamation of contigs and scaffolds spanning the gaps around repetitive regions (Conte and Kocher, 2015; Pootakham et al., 2017). Previously, *P. capsici* was sequenced using diverse sequencing methods (Oxford Nanopore, Illumina HiSeq X Ten), but their N50 length of scaffolds was not comparable to other sequenced genomes (Cui et al., 2019; Reyes-Tena et al., 2019). We leveraged both Illumina HiSeq and PacBio long-read sequencing technologies for a comparative genomics analysis of three *P. capsici* isolates, with an average *de novo* genome assembly size of 75 Mbp. This genome size is comparable with the previously sequenced reference genome LT1534 (64 Mb) (Lamour et al., 2012), but differs from the size of the long-read assembly (110 Mb) (Cui et al., 2019) and Illumina-based assemblies of Mexican isolates (52 Mb) (Reyes-Tena et al., 2019). Considerably fewer scaffolds are present in our assemblies (514 on average) than are present in the reference genome (917), while our genome assembly contained more repeated regions and transposable elements (49% of the genome, on average) than the reference genome. Furthermore, we revealed a comparable number of genes encoding potential secreted effectors in each genome, including RxLR effectors, CRN effectors, and CAZymes which were grouped into six families. Several pathogenicity and necrosis-inducing genes, such as the protease inhibitor, ABC transporter, cytochrome P450, phytotoxin (PcF protein), and NPP1 families (Lamour et al., 2012; Armitage et al., 2018; Yang et al., 2018), were also detected.

Pathogenicity-related genes are often clustered in relatively less conserved, rapidly evolving, gene-poor genome regions containing abundant transposons or repeated elements (Haas et al., 2009; Dutheil et al., 2016; Dale et al., 2019). These genomic regions commonly contain effector genes involved in the evolutionary adaptation of the pathogen to the host (de Jonge et al., 2013; Dale et al., 2019). Rapid evolutionary events in such genomic regions can create isolate-specific or distinct non-core genes that can diversify the different strains of the same species (Dale et al., 2019). Our results also revealed that the pathogenicity-related genes, such as those encoding the CRN and RxLR effectors, are clustered in gene-sparse regions of the genomes. The genome assemblies of the *P. capsici* isolates showed diverse evolutionary trajectories compared with the reference genome, and revealed the multiplicity of the RxLR effectors in the three genomes. We detected more RxLR candidates (average of 220) than were previously identified (73) in a *P. capsici* transcriptome (Lamour et al., 2012). These genes may have played a significant role in the adaptive potential and evolution of the pathogenicity of the *P. capsici* isolates. In contrast, we detected fewer CRN effectors (average of 42) in the three *P. capsici* isolates, which may be due to the different pipeline and prediction methodology used. A total of 84, 60, and 196 CRN effectors were previously predicted in *P. capsici*, *P. ramorum*, and *P. infestans*, respectively (Stam et al., 2013). Differences in structural and functional annotation pipelines might predict the different number of genes in our genome assembly, for example, ABC transporters or proteases.

A gene family clustering analysis facilitated the identification of the common and unique orthologs among the closely and more distantly related pathogen species to study their phylogeny and evolutionary dynamics. The numbers of single-copy orthologous and unique gene clusters in the three *P. capsici* isolates were comparable with the previously reported LT1534 genome (Lamour et al., 2012). Here, we detected an average of 7,877 single-copy orthologs in the three *P. capsici* isolates, which were used for the phylogenetic analysis and to calculate the species evolutionary divergence time. Our phylogenetic topology was consistent with previous reports that *P. cactorum* was closely related to *P. parasitica*, *P. infestans*, and *P. capsici* (Blair et al., 2008; Yang et al., 2018). Additionally, we demonstrated that the less-aggressive isolate (MY-1) is relatively evolutionarily distant from the medium- (JHAI1–7) and highly-aggressive (KPC-7) isolates.

Gene family expansion has frequently been shown to directly or indirectly affect the virulence of oomycete pathogens (Spanu et al., 2010; Schwartze et al., 2014; Morales-Cruz et al., 2015). We observed that a total of 946 and 1,588 gene families in the *P. capsici* clade had undergone expansion and contraction, respectively. More gene families were contracted in the *P. capsici* clade than in the rest of the *Phytophthora* species, although the number of expanded gene families was relatively lower. Our comparative genomic analysis of the three *P. capsici* isolates showed that the expanded genes in the aggressive isolates (KPC-7 and JHAI1–7) were also enriched in the P-loop containing nucleoside triphosphate hydrolase, RxLR effector, nucleoside-triphosphatase activity, hydrolase activity, and DNA binding-related gene families, most of which have been reported to be involved in the pathogenicity of fungal and oomycete pathogens. The expansion of gene families may be caused by the large number of transposons in the *P. capsici* genome (Schwartze et al., 2014; Yang et al., 2018). Nonetheless, more research should be done to interpret the relationship between transposons and gene family expansion.

Recently, genome resequencing has become a useful tool for investigating the genomic basis of the adaptation of oomycete pathogen traits, such as pathogenicity, virulence, fungicide resistance, and host specification (Yang et al., 2018; Zhang et al., 2019). In the present study, the resequencing and SNP calling of the three study isolates (MY-1, JHAI1–7, and KPC-7) and two additional *P. capsici* isolates (Supplementary Table 8) resulted in the detection of average 1,023,437 SNPs in comparison with the previously sequenced LT1534 reference genome. Many disruptive variations were observed in ABC transporter-encoding genes, which was also confirmed in the gene family expansion analysis. It indicated that high nucleotide substitutions were accumulated during adaptive evolution, but their heterozygosity levels were not significantly different from other gene families (Figure 5D). Our findings may serve as stable markers and variants of pathogenicity-related genes consistent with the recent report of polymorphism among the *avirulence* (*Avr*) genes of 29 *P. sojae* isolates (Zhang et al., 2019). These polymorphic variations among *P. capsici* isolates require further investigation, however.

Pathogenicity-related genes are usually affected by selective pressure, which was found to have driven nucleotide substitutions in these genes in the three *P. capsici* isolates. Moreover, consistent with the “two-speed genome” theory, our findings supported the hypothesis that the pathogenicity-related genes in oomycete pathogens are located in highly repetitive gene-sparse regions to enable them to evolve rapidly to adapt to host changes (Dong et al., 2015). Our analysis also revealed that the expansion and nucleotide variations of some gene families, such as the ABC transporters (PHI:1018_2042), CRN (PHI:656), and RxLR (PHI:530) effectors corresponded to the isolate aggressiveness profiles, with a greater expansion in more highly aggressive isolates. Among them, the ABC transporter (Ammar et al., 2013), CRN (Torto et al., 2003), and RxLR (Shan et al., 2004) families are known as virulence determinants in plant pathogens. Furthermore, the genomic contents of these gene families were also consistent with the two genome evolutionary theory for high-repeat elements and nucleotide substitutions. These genomic changes provided further evidence that the aggressiveness of the *P. capsici* isolates might have evolved based on diverse mechanisms, two-speed genome theory, accumulation of disruptive variants, and extensive matings and crossover between *P. capsici* isolates or within genomes.

To summarize, we sequenced and compared the genomes of three *P. capsici* isolates with diverse aggressiveness levels using long- and short-read sequencing technologies. Our analysis revealed that the less aggressive isolate was distantly related to the more aggressive isolates. Furthermore, we detected gene family expansions in the pathogenicity-related genes, effectors and ABC transporters, in the aggressive isolates. The resequencing of these *P. capsici* isolates revealed striking genomic differences from the reference genome LT1534. It revealed that more disruptive substitutions occurred in pathogenicity-related genes, whose gene families were expanded during evolutionary process. Our results elucidate the genomic similarities and differences among the less and highly aggressive isolates, and establish a foundation for further research to analyze the genomic and sequence variations for the functional study of pathogenicity-related genes.

## Data Availability Statement

The dataset generated for this study can be found in the NCBI database (SAMN17101195-7).

## Author Contributions

MIS, J-KK, and B-CK directed the project. J-HL and MIS analyzed the data and performed the experiments. J-HL, MIS, and B-CK wrote the manuscript. All authors contributed to the article and approved the submitted version.

## Conflict of Interest

The authors declare that the research was conducted in the absence of any commercial or financial relationships that could be construed as a potential conflict of interest.

## Publisher’s Note

All claims expressed in this article are solely those of the authors and do not necessarily represent those of their affiliated organizations, or those of the publisher, the editors and the reviewers. Any product that may be evaluated in this article, or claim that may be made by its manufacturer, is not guaranteed or endorsed by the publisher.

## Footnotes

1
http://www.repeatmasker.org/RepeatModeler/

2
http://www.repeatmasker.org/

3
https://www.uniprot.org/database/DB-0072

4
http://www.timetree.org/

## Supplementary Material

Supplementary Figure 1 Appearance of disease symptoms and plant mortality. The susceptible control pepper variety “Tean” was inoculated with three *P. capsici* isolates, KPC-7, JHAI1–7, and MY-1. The disease symptom development and disease severity were recorded from 0 to 21 days after inoculation.

Supplementary Figure 2 Mycelium growth of three isolates on two different grown media. **(A–C)** Growth of isolates on V8 agar. **(D–F)** Growth of isolates on potato dextrose agar.

Supplementary Figure 3 *k*-mer distribution plot of the three *P. capsici* isolates.

Supplementary Figure 4 Scatterplot analysis of the gene density distribution. Graphical result for **(A)** MY-1, **(B)** JHAI1–7, and **(C)** KPC-7. All distances were transformed to log_2_ values for visualization. The colored bar for gene density represents the number of genes in a position/the number of total input genes.

Supplementary Figure 5 Dot plot analysis. Dot plot analysis comparing the published *P. capsici* reference genome and the *de novo* assembled genomes in the present study. The *x*-axis represents the *P. capsici* strain LT1534 genome version 11.0. The *y*-axis represents each strain, **(A)** JHAI1–7, **(B)** KPC-7, or **(C)** MY-1.

Supplementary Figure 6 The number of CAZymes detected in this study. **(A)** Number of CAZymes in four isolates. **(B)** Number of CAZymes including signal peptides in four isolates. Signal peptides were predicted using SignalP5.0 program with default option. GH, glycosyl hydrolase; GT, glycosyl transferase; PL, polysaccharide lyases; AA, auxiliary activities; CBM, carbohydrate-binding modules; CE, carbohydrate esterases.

Supplementary Figure 7 Scatterplot analysis of the cytoplasmic effector gene density distribution. **(A–F)** Graphical result for the RxLR effectors in **(A)** MY-1, **(B)** JHAI1–7, and **(C)** KPC-7, as well as the CRN effectors in **(D)** MY-1, **(E)** JHAI1–7, and **(F)** KPC-7. All distances were transformed to log_2_ values for visualization. The color bar for gene density represents the number of genes in a position/the number of total input genes.

Supplementary Figure 8 Raw data for the gene family expansion and contraction evaluation performed using CAFE. “+” indicates the number of expanded gene families and “–” indicates the number of contracted gene families. Parenthesis represents significantly expanded/contracted gene families and used for a downstream analysis.

Supplementary Figure 9 Nucleotide variations located in the region for mating type locus of *P. capsici* (Lamour et al., 2012). Red colors represents the previously described SNPs which convert the mating type of *P. capsici*. Two correlating SNPs with the previous reports are highlighted by dotted line. R, reference alleles; A, alternative alleles.

Supplementary Figure 10 Representative SNP patterns of RxLR, CRN, and ABC transporter coding genes. R, reference alleles; A, alternative alleles; A′, second alternative alleles.

Supplementary Table 1 Detailed information about the *P. capsici* isolates used in the present study.

Supplementary Table 2 Summary of the reads generated in the present study.

Supplementary Table 3 Summary of the repeat elements in *de novo* assembled genomes of the three *P. capsici* isolates.

Supplementary Table 4 Positively selected orthologous genes.

Supplementary Table 5 Top 20 InterPro (IPR) terms associated with the significantly expanded/contracted gene families in the *P. capsici*-specific node (rectangle node in Figure 4).

Supplementary Table 6 Enriched Gene Ontology (GO) terms associated with the significantly expanded/contracted gene families in the *P. capsici*-specific node (rectangle node in Figure 4).

Supplementary Table 7 InterPro (IPR) terms associated with the expanded and contracted gene families in the node containing the high- and low-aggressiveness isolates.

Supplementary Table 8 Gene ontology (GO) terms associated with the expanded and contracted gene families in the node containing the high- and low-aggressiveness isolates.

Supplementary Table 9 Summary of resequencing statistics.

Supplementary Table 10 Enriched Gene ontology (GO) terms for the genes containing more than 20 disruptive variants.

Supplementary Data 1 SNP calling results of five *P. capsici* isolates.

Supplementary Data 2 Functional and structural annotation results of *P. capsici* isolates, KPC-7, JHAI1–7, and MY-1.
