# Supplementary material for: Comparative Genomic Analysis Reveals Genetic Variation and Adaptive Evolution in the Pathogenicity-Related Genes of Phytophthora capsici
Source: Front Microbiol. 2021 Aug 13;12:694136. doi: 10.3389/fmicb.2021.694136 (PMC8415033; doi:10.3389/fmicb.2021.694136)
Supplement: Supplementary Figure 1 — Appearance of disease symptoms and plant mortality. The susceptible control pepper variety “Tean” was inoculated with three P. capsici isolates, KPC-7, JHAI1–7, and MY-1. The disease symptom development and disease severity were recorded from 0 to 21 days after inoculation. [file Presentation_1.pdf]

SUPPLEMENTARY FIGURES

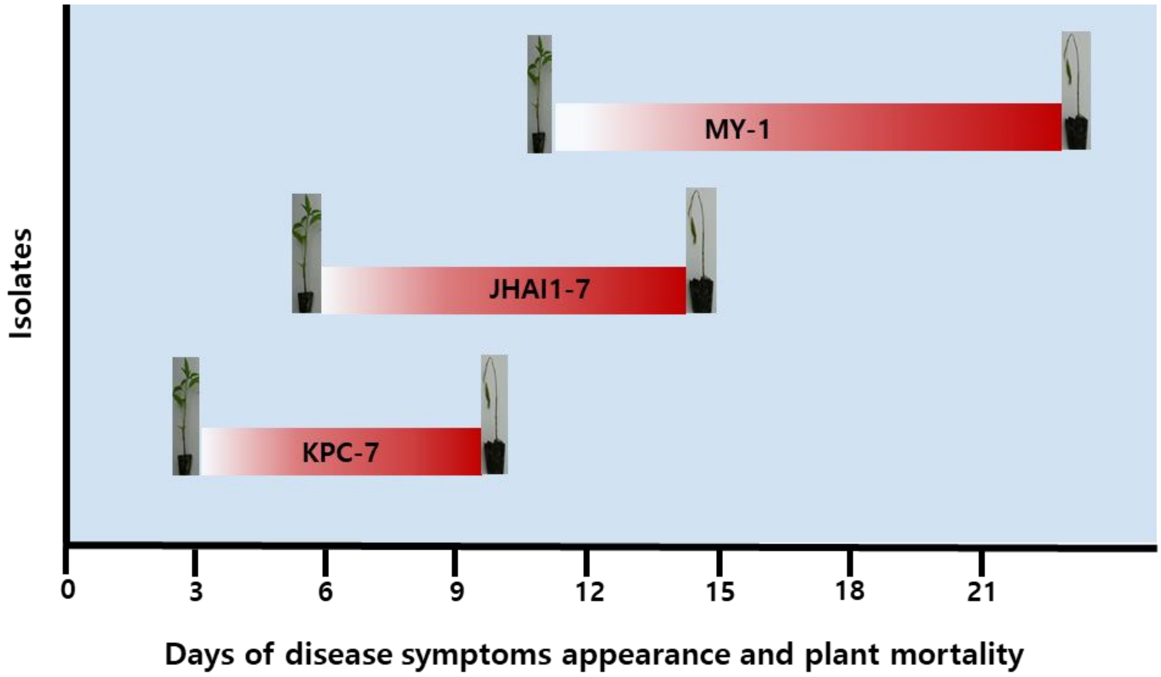

Figure S1 Appearance of disease symptoms and plant mortality. The susceptible control pepper variety 'Tean' was inoculated with three *P. capsici* isolates, KPC-7, JHAI-7, and MY-1. The disease symptom development and disease severity were recorded from 0–21 days after inoculation.

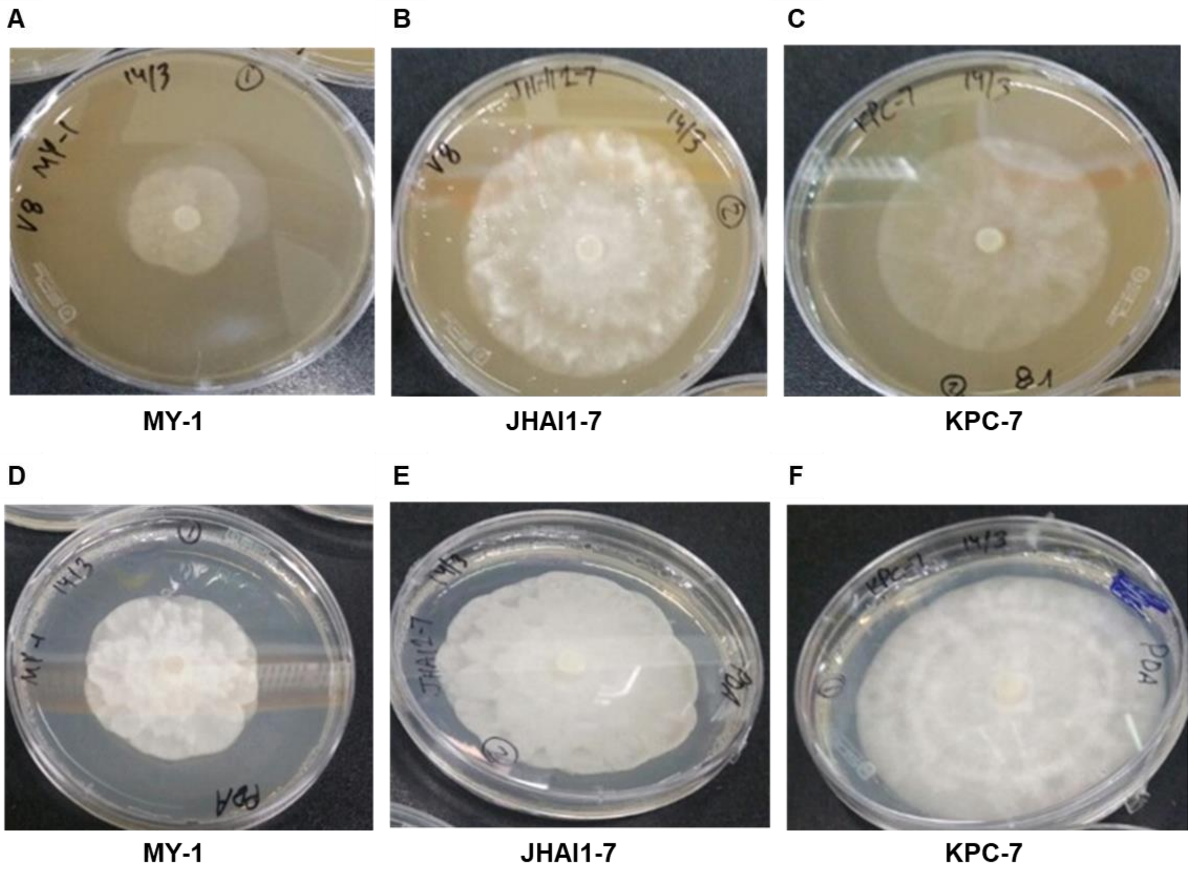

Figure S2 Mycelium growth of three isolates on two different grown media. (A, B, C) Growth of isolates on V8 agar. (D, E, F) Growth of isolates on potato dextrose agar.

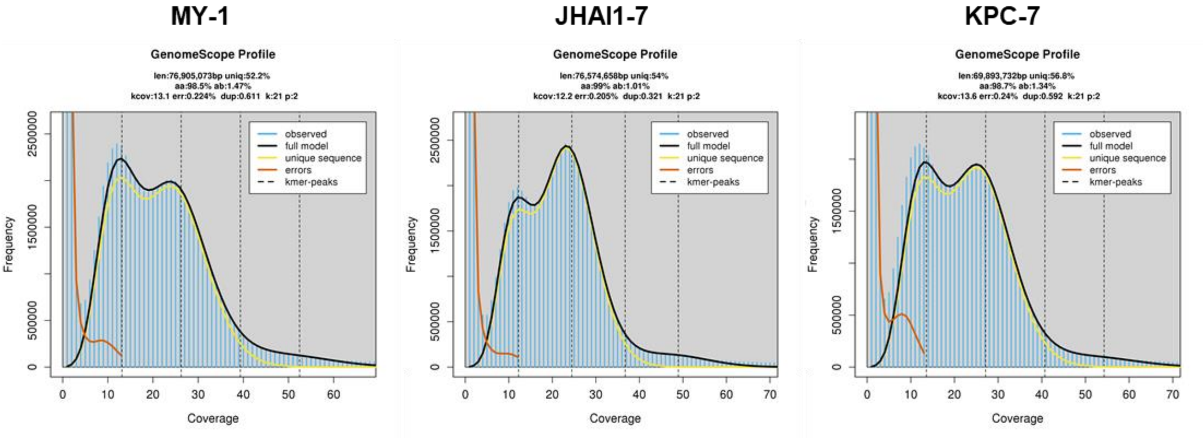

Figure S3 *k*-mer distribution plot of the three *P. capsici* isolates.

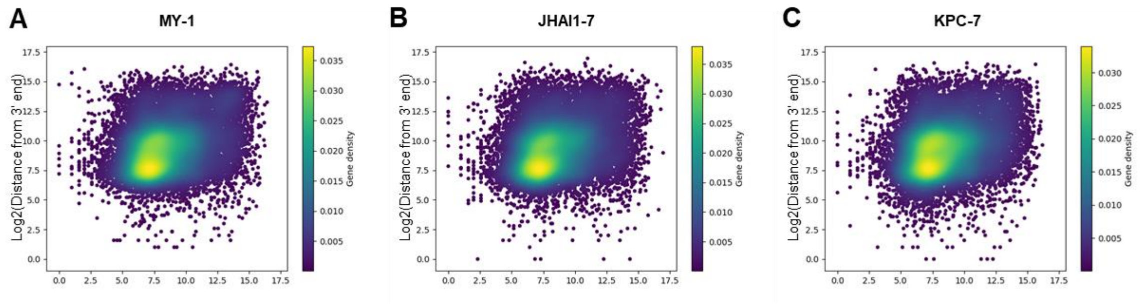

Figure S4 Scatterplot analysis of the gene density distribution. Graphical result for (A) MY-1, (B) JHAI1-7, and (C) KPC-7. All distances were transformed to $\log_2$ values for visualization. The colored bar for gene density represents the number of genes in a position/the number of total input genes.

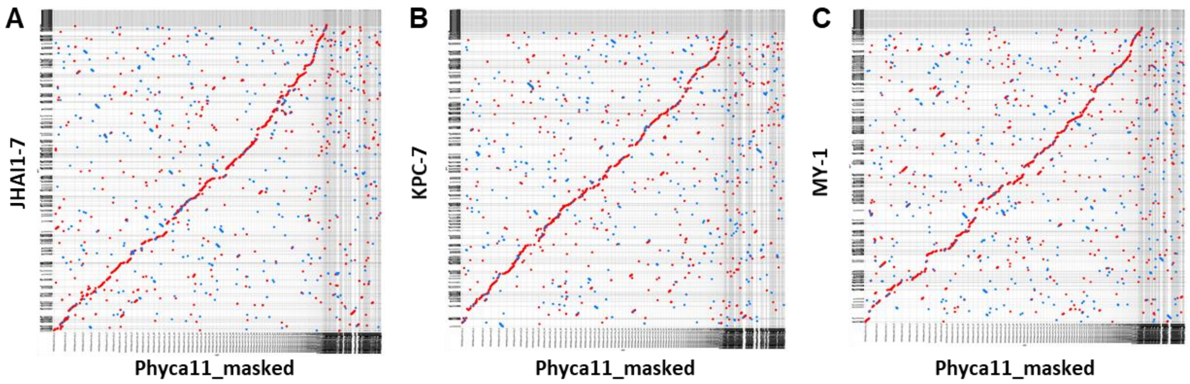

Figure S5 Dot plot analysis. Dot plot analysis comparing the published *P. capsici* reference genome and the *de novo* assembled genomes in the present study. The x-axis represents the *P. capsici* strain LT1534 genome version 11.0. The y-axis represents each strain, (A) JHAI1-7, (B) KPC-7, or (C) MY-1.

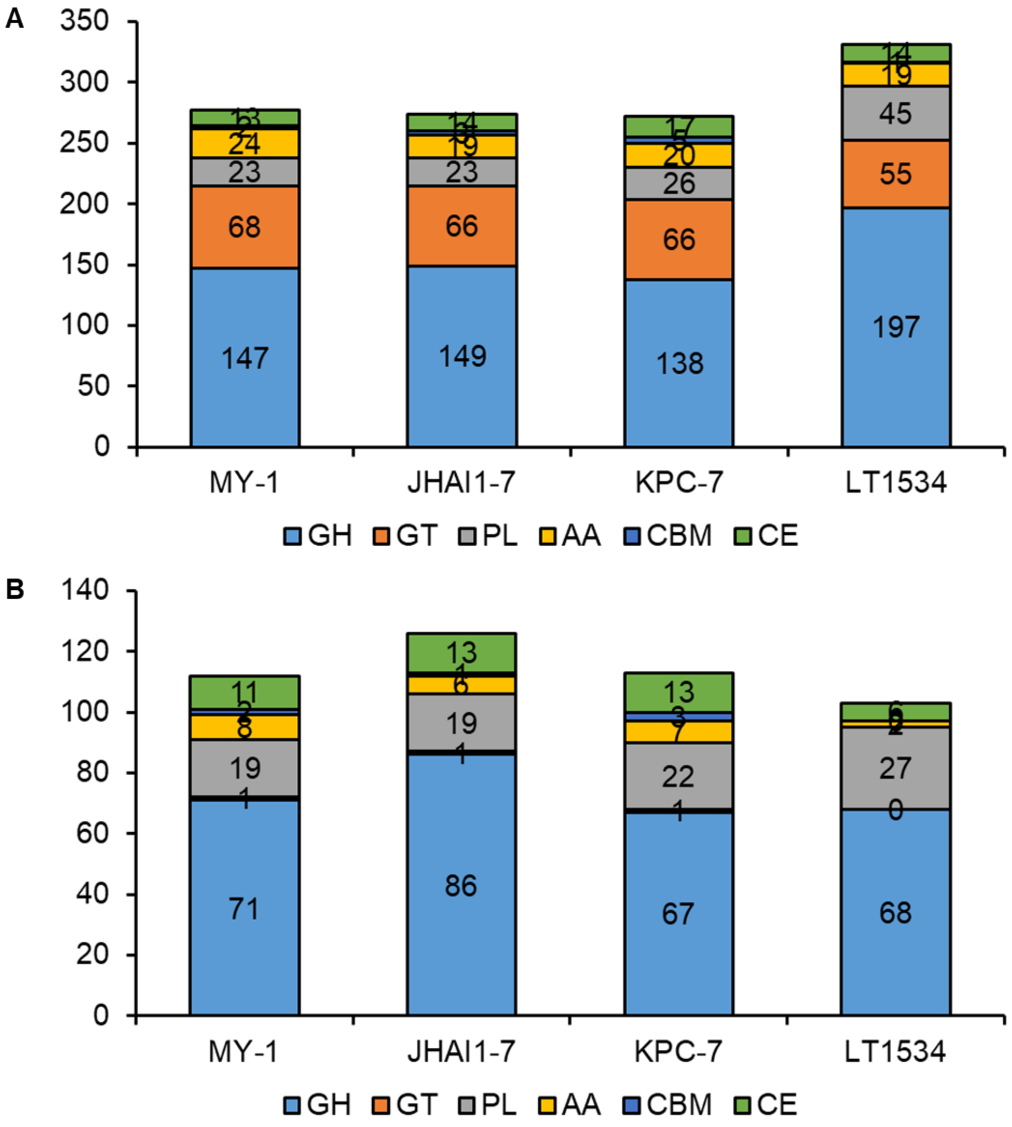

Figure S6 The number of CAZymes detected in this study. (A) Number of CAZymes in four isolates. (B) Number of CAZymes including signal peptides in four isolates. Signal peptides were predicted using SignalP5.0 program with default option. GH: Glycosyl hydrolase; GT: Glycosyl transferase; PL: Polysaccharide lyases; AA: Auxiliary activities; CBM: Carbohydrate-binding modules; CE: Carbohydrate esterases.

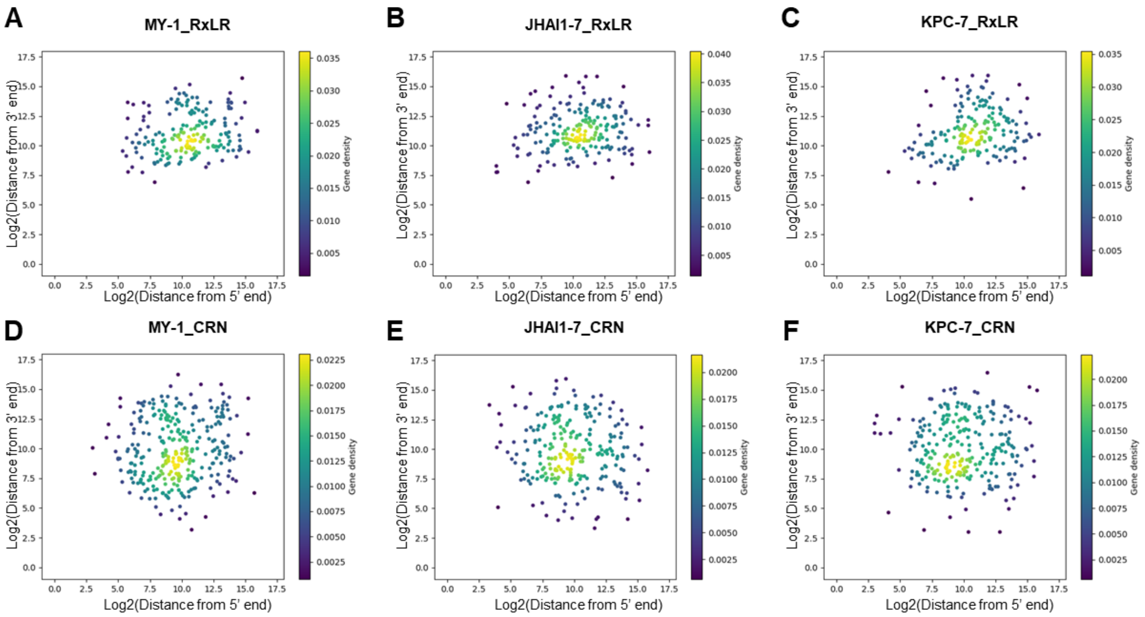

Figure S7 Scatterplot analysis of the cytoplasmic effector gene density distribution. (A–F) Graphical result for the RxLR effectors in (A) MY-1, (B) JHAI1-7, and (C) KPC-7, as well as the CRN effectors in (D) MY-1, (E) JHAI1-7, and (F) KPC-7. All distances were transformed to $\log_2$ values for visualization. The color bar for gene density represents the number of genes in a position/the number of total input genes.

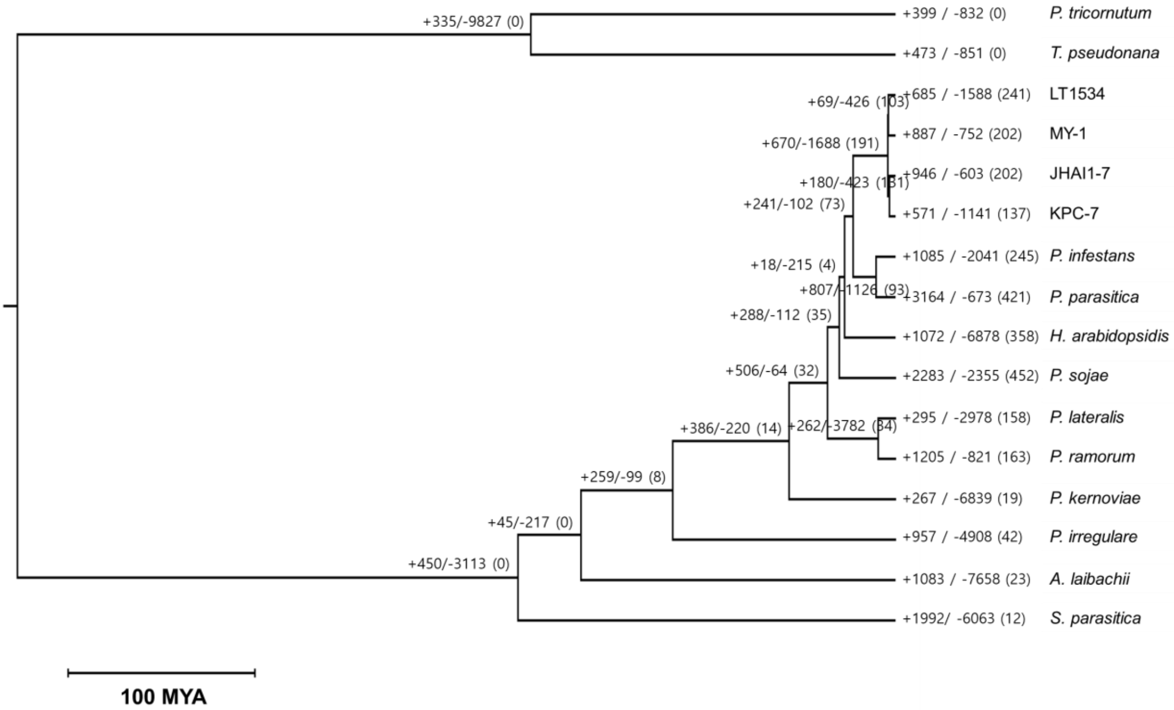

Figure S8 Raw data for the gene family expansion and contraction evaluation performed using CAFE. '+' indicates the number of expanded gene families and '–' indicates the number of contracted gene families. Parenthesis represents significantly expanded/contracted gene families and used for a downstream analysis.

Mating type	M2	M1	M1	M1	M1
**Marker**	**MY-1**	**JHAI1-7**	**KPC-7**	**PEP**	**Pc038**
PHYCAscaffold_4_875573	RA	RA	RA	RA	AA
PHYCAscaffold_4_877292	RA	RA	RA	RA	AA
PHYCAscaffold_4_877466	RA	RA	RA	RA	AA
PHYCAscaffold_4_880664	RA	RA	RA	RA	AA
PHYCAscaffold_4_905925	RA	RA	RA	RA	AA
PHYCAscaffold_4_905926	RA	RA	RA	RA	AA
PHYCAscaffold_4_906310	RA	RA	RA	RA	AA
PHYCAscaffold_4_907167	RR	RA	RA	RA	AA
PHYCAscaffold_4_907269	RR	RA	RA	RA	AA
PHYCAscaffold_4_907278	RR	RA	RA	RA	AA
PHYCAscaffold_4_907338	RR	RA	RA	RA	AA
PHYCAscaffold_4_907371	RR	RA	RA	RA	AA
PHYCAscaffold_4_907662	RR	RA	RA	RA	AA
PHYCAscaffold_4_907692	RR	RA	RA	RA	AA
PHYCAscaffold_4_907935	RR	RA	RA	RA	AA
PHYCAscaffold_4_908349	RA	AA	AA	RA	AA
PHYCAscaffold_4_908442	RA	AA	AA	RA	AA

Figure S9 Nucleotide variations located in the region for mating type locus of *P. capsici* (Lamour et al., 2012). Red colors represents the previously described SNPs which convert the mating type of *P. capsici*. Two correlating SNPs with the previous reports are highlighted by dotted line. R: Reference alleles; A: Alternative alleles.

PcAvh103					
Marker	MY-1	JHAI1-7	KPC-7	Pc038	PEP
PHYCAscaffold_52:143949	RA	AA	AA	AA	RA
PHYCAscaffold_52:143955	RR	RA	RA	AA	RR
PHYCAscaffold_52:144122	RA	RA	RA	RR	RA
PHYCAscaffold_52:144139	RA	RR	RR	RR	RA
PHYCAscaffold_52:144154	RA	RR	RR	RR	RA
PHYCAscaffold_52:144163	RA	RR	RR	RR	RA
PHYCAscaffold_52:144191	RA	AA	AA	AA	RA
PHYCAscaffold_52:144200	RA	AA	AA	AA	RA
PHYCAscaffold_52:144219	RR	RA	RA	AA	RR
PHYCAscaffold_52:144239	RA	AA	AA	AA	RA
PHYCAscaffold_52:144242	RA	AA	AA	AA	RA
PHYCAscaffold_52:144273	RA	AA	AA	AA	RA
PHYCAscaffold_52:144276	RA	AA	AA	AA	RA
PHYCAscaffold_52:144287	RR	RA	RA	AA	RR

PcCRN4					
Marker	MY-1	JHAI1-7	KPC-7	Pc038	PEP
PHYCAscaffold_21:181970	RR	AA	RA	RR	RA
PHYCAscaffold_21:181971	RR	AA	RA	RR	RA
PHYCAscaffold_21:181993	RR	RR	RA	RR	RA
PHYCAscaffold_21:182006	RR	AA	RR	RR	RR
PHYCAscaffold_21:182033	RR	RR	RA	RR	RA
PHYCAscaffold_21:182053	RR	RR	RA	RR	RA
PHYCAscaffold_21:182075	RR	RR	RA	RR	RA
PHYCAscaffold_21:182076	RR	AA	RA	AA	RA
PHYCAscaffold_21:182120	RR	AA	RA	AA	RA
PHYCAscaffold_21:182225	RR	RR	RA	AA	RA
PHYCAscaffold_21:182250	RR	AA	RR	RR	RR
PHYCAscaffold_21:182275	RR	AA	RA	RR	RA
PHYCAscaffold_21:182303	RR	AA	RA	RR	RA
PHYCAscaffold_21:182486	RR	RR	RR	AA	RR
PHYCAscaffold_21:182605	RR	RR	RA	RR	RA
PHYCAscaffold_21:182612	RR	RR	RA	RR	RA
PHYCAscaffold_21:182707	RR	RR	RR	AA	RR
PHYCAscaffold_21:182712	RR	AA	RR	RR	RR
PHYCAscaffold_21:182739	RR	AA	RR	RR	RR
PHYCAscaffold_21:182740	RR	RR	RA	RR	RA
PHYCAscaffold_21:182750	RR	AA	**RA'**	**A'A'**	**RA'**
PHYCAscaffold_21:182900	RR	AA	RR	RR	RR
PHYCAscaffold_21:182984	RR	AA	RA	RR	RA
PHYCAscaffold_21:183031	RR	AA	RR	RR	RR
PHYCAscaffold_21:183043	RR	AA	RR	RR	RR
PHYCAscaffold_21:183254	RR	RR	AA	RR	RA
PHYCAscaffold_21:183546	RR	AA	RA	AA	RA
PHYCAscaffold_21:183554	AA	AA	RA	AA	RA
PHYCAscaffold_21:183629	AA	AA	RA	AA	RA
PHYCAscaffold_21:183641	AA	RR	RR	RR	RR

ABC_PhyCa11_partial					
Marker	MY-1	JHA1-7	KPC-7	Pc038	PEP
PHYCAscaffold_15:660480	RA	RR	RR	RR	RA
PHYCAscaffold_15:660495	RR	AA	AA	AA	RA
PHYCAscaffold_15:660588	RA	RR	RR	RR	RA
PHYCAscaffold_15:660606	RA	RR	RR	RR	RR
PHYCAscaffold_15:660633	RA	RR	RR	RR	RR
PHYCAscaffold_15:660660	RA	RR	RR	RR	RR
PHYCAscaffold_15:660720	RR	RR	RR	RR	RA
PHYCAscaffold_15:660728	RR	RR	RR	RR	RA
PHYCAscaffold_15:660750	RA	AA	AA	AA	AA
PHYCAscaffold_15:660783	RA	AA	AA	AA	RA
PHYCAscaffold_15:660819	RR	RR	RR	RR	RA
PHYCAscaffold_15:660828	RA	AA	AA	AA	RA
PHYCAscaffold_15:660834	RA	AA	AA	AA	RA
PHYCAscaffold_15:660844	RA	AA	AA	AA	RA
PHYCAscaffold_15:660858	RR	RR	RR	RR	RA
PHYCAscaffold_15:660864	RA	AA	AA	AA	RA
PHYCAscaffold_15:660900	RA	AA	AA	AA	RA
PHYCAscaffold_15:660998	RA	RR	RR	RR	RA
PHYCAscaffold_15:661125	RA	AA	AA	AA	RA
PHYCAscaffold_15:661137	RR	RR	RR	RR	RA
PHYCAscaffold_15:661310	AA	RR	RR	RR	RA
PHYCAscaffold_15:661484	RA	RR	RR	RR	RA
PHYCAscaffold_15:661619	RR	RR	RR	RR	RA
PHYCAscaffold_15:661667	RA	AA	AA	AA	RA
PHYCAscaffold_15:661688	RA	AA	AA	AA	RA
PHYCAscaffold_15:661700	RR	RR	RR	RR	RA
PHYCAscaffold_15:661703	RR	AA	AA	AA	RA
PHYCAscaffold_15:661724	RR	AA	AA	AA	RA
PHYCAscaffold_15:661727	RR	AA	AA	AA	RA
PHYCAscaffold_15:661778	RR	AA	AA	AA	RA
PHYCAscaffold_15:661802	RR	RR	RR	RR	RA
PHYCAscaffold_15:661811	AA	AA	AA	AA	RA
PHYCAscaffold_15:661868	AA	AA	AA	AA	RA
PHYCAscaffold_15:661886	RR	AA	AA	AA	RA
PHYCAscaffold_15:661904	RA	RR	RR	RR	RA
PHYCAscaffold_15:661949	RA	RR	RR	RR	RA
PHYCAscaffold_15:661985	AA	AA	AA	AA	RA

Figure S10 Representative SNP patterns of RxLR, CRN, and ABC transporter coding genes. R: Reference alleles; A: Alternative alleles; A': Second alternative alleles.
