# Supplementary material for: Comparative Genomic Analysis Reveals Genetic Variation and Adaptive Evolution in the Pathogenicity-Related Genes of Phytophthora capsici
Source: Front Microbiol. 2021 Aug 13;12:694136. doi: 10.3389/fmicb.2021.694136 (PMC8415033; doi:10.3389/fmicb.2021.694136)
Supplement: Supplementary Table 1 — Detailed information about the P. capsici isolates used in the present study. [file Table_1.pdf]

SUPPLEMENTARY TABLES

Table S1 Detailed information about the *P. capsici* isolates used in the present study.

Isolate	Collection site	Host	Aggressiveness	Mating type	Source	References
KPC-7	Kangwon/Korea	*C. annuum*	High	A1	KRICT	Jo et al. (2014)
JHAI1-7	Chungbuk/Korea	*C. annuum*	Moderate	A1	KRICT	Liu et al. (2014)
MY-1	Kangwon/Korea	*C. annuum*	Low	A2	KRICT	Jo et al. (2014)
Pc038	Miryang/Korea	*C. annuum*	High	A1	KNU	Abebe et al. (2016)
PEP	Chiang Mai/Thailand	*C. annuum*	High	A1	EWS	(Unpublished)

Table S2 Summary of the reads generated in the present study.

	**MY-1**	**JHA1-7**	**KPC-7**	**Pc038**	**PEP**
No. of total HiSeq reads	28,853,066	28,240,964	27,025,390	27,401,702	33,830,928
GC(%) of HiSeq reads	48,128	49,763	48,896	49,953	49,639
Q20(%) of HiSeq reads	94.073	94.444	95.082	94.788	95.091
No. of total Sequel sub-reads	1,356,081	1,248,011	1,351,163	-	-
N50 of Sequel sub-reads	13,093	13,367	19,000	-	-
Average Sequel sub-read length	7,391	6,950	7,319	-	-

Table S3 Summary of the repeat elements in *de novo* assembled genomes of the three *P. capsici* isolates.

	MY-1			JHAI1-7			KPC-7		
	No. of elements	length occupied (bp)	% of sequences	No. of elements	length occupied (bp)	% of sequences	No. of elements	length occupied (bp)	% of sequences
**SINEs**	**611**	**597,867**	**0.78**	**501**	**812,239**	**1.05**	**523**	**670,689**	**0.8948**
ALUs	0	0	0	0	0	0	0	0	0
MIRs	0	0	0	0	0	0	0	0	0
**LINEs**	**587**	**920,261**	**1.2**	**627**	**683,359**	**0.88**	**909**	**818,305**	**1.0917**
LINE1	233	139,085	0.18	292	119,611	0.15	326	229,647	0.3064
LINE2	0	0	0	0	0	0	0	0	0
L3/CR1	0	0	0	0	0	0	0	0	0
**LTR elements**	**8,742**	**17,474,509**	**22.8**	**7,997**	**16,869,652**	**21.95**	**8,183**	**16,627,734**	**22.18**
ERVL	0	0	0	0	0	0	0	0	0
ERVL-MaLRs	0	0	0	0	0	0	0	0	0
ERV_classI	0	0	0	1	379	0	0	0	0
ERV_class II	0	0	0	0	0	0	0	0	0
**DNA elements**	**8,249**	**5,417,825**	**7.07**	**7124**	**4,589,375**	**5.97**	**7493**	**4,484,685**	**5.9831**
hAT-Charlie	25	12,861	0.01	0	0	0	0	0	0
TcMar-Tigger	0	0	0	0	0	0	0	0	0
**Unclassified**	**10,768**	**5,358,527**	**6.99**	**11325**	**6,693,472**	**8.71**	**12,125**	**5,951,230**	**7.9396**
**Total interspersed repeats**		**29,768,989**	**38.85**		**29,648,097**	**38.58**		**28,552,643**	**38.0926**
**Small RNA**	**58**	**185,128**	**0.24**	**92**	**400,813**	**0.52**	**82**	**188,149**	**0.251**
**Satellites**	**0**	**0**	**0**	**63**	**50,251**	**0.06**	**4**	**332**	**0.0004**
**Simple repeats**	**4,436**	**248,469**	**0.32**	**4519**	**284,814**	**0.37**	**4396**	**266,630**	**0.3557**
**Low complexity**	**516**	**27,018**	**0.03**	**532**	**27,947**	**0.03**	**490**	**26,046**	**0.0347**

Table S4 Positively selected orthologous genes.

No.	PFAM domain structure			omega	Function in SwissProt
	**KPC-7**	**JHA1-7**	**MY1**		
1	MIF4G (KPC00002041.1)	MIF4G (JHA00005295.1)	MIF4G (MY100013316.1)	3.662	Eukaryotic translation initiation factor 4G
2	HTH_Tnp_Tc5 (KPC00013468.1)	DDE_1 (JHA00006316.1)	HTH_Tnp_Tc5 (MY100013869.1)	1.793	Pogo transposable element with KRAB domain
3	RXLR (KPC00005514.1)	- (JHA00003936.1)	- (MY100008048.1)	1.741	RxLR effector protein PITG_15679
4	- (KPC00011916.1)	- (JHA00009134.1)	- (MY100011780.1)	1.627	MAM and LDL-receptor class A domain-containing protein 2
5	- (KPC00008358.1)	- (JHA00009044.1)	- (MY100001585.1)	1.612	DNA-directed RNA polymerase III subunit rpc1
6	ORC4_C (KPC00014271.1)	- (JHA00011388.1)	- (MY100010547.1)	1.506	Origin recognition complex subunit 4
7	adh_short (KPC00010360.1)	adh_short (JHA00003350.1)	adh_short (MY100002630.1)	1.429	Uncharacterized oxidoreductase SSP1627
8	- (KPC00000597.1)	- (JHA00010824.1)	- (MY100014829.1)	1.411	Lysine-specific demethylase 3A-A
9	- (KPC00011547.1)	- (JHA00010575.1)	- (MY100004045.1)	1.378	Putative glutamate--cysteine ligase 2
10	Gemin6 (KPC00005425.1)	Gemin6 (JHA00009392.1)	Gemin6 (MY100005440.1)	1.249	Tyrosine--tRNA ligase
11	- (KPC00002003.1)	- (JHA00001409.1)	- (MY100006425.1)	1.173	DnaJ homolog subfamily C member 14
12	MYCBPAP; PGK (KPC00011882.1)	MYCBPAP; PGK (JHA00002390.1)	MYCBPAP (MY100008470.1)	1.148	MYCBP-associated protein
13	- (KPC00002974.1)	- (JHA00001180.1)	- (MY100011140.1)	1.138	Phospholipid scramblase 3
14	- (KPC00004638.1)	- (JHA00010016.1)	- (MY100001738.1)	1.129	Ribosome-binding factor A
15	EF-hand_5 (KPC00005388.1)	- (JHA00007663.1)	EF-hand_5 (MY100002868.1)	1.11	Lysophosphatidylcholine acyltransferase 2B
16	HSBP1 (KPC00002281.1)	HSBP1 (JHA00002968.1)	HSBP1 (MY100007860.1)	1.098	Heat shock factor-binding protein 1
17	- (KPC00008993.1)	- (JHA00003480.1)	- (MY100009830.1)	1.067	F-box/LRR-repeat protein 5
18	- (KPC00005845.1)	- (JHA00009954.1)	- (MY100008975.1)	1.066	Dopamine beta-hydroxylase
19	- (KPC00003574.1)	- (JHA00000627.1)	- (MY100011975.1)	1.053	Isoleucine--tRNA ligase
20	- (KPC00001900.1)	- (JHA00014595.1)	- (MY100014303.1)	1.038	DNA-directed RNA polymerase subunit beta
21	- (KPC00013640.1)	- (JHA00004967.1)	- (MY100013393.1)	1.027	Dimethyladenosine transferase 1, mitochondrial
22	- (KPC00008988.1)	- (JHA00003486.1)	- (MY100009836.1)	1.017	Helicase ARIP4
23	- (KPC00013005.1)	- (JHA00009117.1)	- (MY100011796.1)	1.012	-
24	MtN3_slv (KPC00005803.1)	MtN3_slv (JHA00009212.1)	MtN3_slv (MY100010167.1)	1.012	Bidirectional sugar transporter SWEET11
25	- (KPC00007669.1)	- (JHA00004477.1)	- (MY100012077.1)	1.01	UPF0053 protein BUsg_314
26	- (KPC00010345.1)	- (JHA00003367.1)	- (MY100002608.1)	1.009	Protein FdhE homolog

Table S5 Top 20 InterPro (IPR) terms associated with the significantly expanded/contracted gene families in the *P. capsici*-specific node (Rectangle node in Figure 4).

Node	No.	IPR code	Function	Count
P. capsici specific Node	1	IPR027417	P-loop containing nucleoside triphosphate hydrolase	214
	2	IPR031825	RXLR phytopathogen effector protein	139
	3	IPR036770	Ankyrin repeat-containing domain superfamily	95
	4	IPR010285	DNA helicase Pif1-like	79
	5	IPR020683	Ankyrin repeat-containing domain	78
	6	IPR027806	Harbinger transposase-derived nuclease domain	69
	7	IPR012337	Ribonuclease H-like superfamily	62
	8	IPR002110	Ankyrin repeat	59
	9	IPR025476	Helitron helicase-like domain	43
	10	IPR004875	DDE superfamily endonuclease domain	41
	11	IPR018289	MULE transposase domain	36
	12	IPR016181	Acyl-CoA N-acyltransferase	30
	13	IPR000182	GNAT domain	30
	14	IPR038765	Papain-like cysteine peptidase superfamily	30
	15	IPR010530	NADH-ubiquinone reductase complex 1 MLRQ subunit	27
	16	IPR007527	Zinc finger, SWIM-type	27
	17	IPR005821	Ion transport domain	24
	18	IPR006600	HTH CenpB-type DNA-binding domain	20
	19	IPR036589	Homocysteine-binding domain superfamily	19
	20	IPR003726	Homocysteine-binding domain	19

Table S6 Enriched Gene Ontology (GO) terms associated with the significantly expanded/contracted gene families in the *P. capsici*-specific node (Rectangle node in Figure 4).

Node	No.	Term_ID	description	Type	FDR
P. Capsici specific Node	1	GO:0004386	helicase activity	MF	4.20E-30
	2	GO:0017111	nucleoside-triphosphatase activity	MF	9.80E-10
	3	GO:0016817	hydrolase activity, acting on acid anhydrides	MF	1.80E-09
	4	GO:0016787	hydrolase activity	MF	0.00065
	5	GO:0003676	nucleic acid binding	MF	0.015
	6	GO:0016853	isomerase activity	MF	0.026
	1	GO:0006259	DNA metabolic process	BP	1.40E-27
	2	GO:0042592	homeostatic process	BP	2.30E-25
	3	GO:0065008	regulation of biological quality	BP	8.10E-24
	4	GO:0006996	organelle organization	BP	3.30E-23
	5	GO:0006950	response to stress	BP	1.10E-22
	6	GO:0050896	response to stimulus	BP	1.30E-20
	7	GO:0065007	biological regulation	BP	3.60E-12
	8	GO:0006139	nucleobase-containing compound metabolic process	BP	7.80E-07
	9	GO:0006807	nitrogen compound metabolic process	BP	0.0013
	10	GO:0006457	protein folding	BP	0.0086
	11	GO:0043170	macromolecule metabolic process	BP	0.032

Table S7 InterPro (IPR) terms associated with the expanded and contracted gene families in the node containing the high- and low-aggressive isolates.

Type	Rank	IPR code	Function	No. of IPR codes
Contracted in low-aggressive isolates	1	IPR012337	Ribonuclease H-like superfamily	15
	2	IPR036291	NAD(P)-binding domain superfamily	14
	3	IPR002347	Short-chain dehydrogenase/reductase SDR	14
	4	IPR036397	Ribonuclease H superfamily	10
	5	IPR036871	PX domain superfamily	9
	6	IPR001683	Phox homologous domain	9
	7	IPR000477	Reverse transcriptase domain	8
	8	IPR027417	P-loop containing nucleoside triphosphate hydrolase	6
	9	IPR002156	Ribonuclease H domain	6
	10	IPR003439	ABC transporter-like	4
Expanded in low-aggressive isolates	1	IPR010089	Flavoprotein WrbA-like	30
	2	IPR008254	Flavodoxin/nitric oxide synthase	30
	3	IPR029039	Flavoprotein-like superfamily	30
	4	IPR005025	NADPH-dependent FMN reductase-like	30
	5	IPR010530	NADH-ubiquinone reductase complex 1 MLRQ subunit	27
	6	IPR007817	Pyoverdine/dityrosine biosynthesis protein	15
	7	IPR042098	Taurine dioxygenase TauD-like superfamily	12
	8	IPR003819	TauD/TfdA-like domain	12
	9	IPR012337	Ribonuclease H-like superfamily	12
	10	IPR008906	HAT, C-terminal dimerisation domain	10
Contracted in high-aggressive isolatesn high-aggressiveness levels of isolates aggressiveness levels of	1	IPR001938	Thaumatin family	13
	2	IPR037176	Osmotin/thaumatin-like superfamily	13
	3	IPR016024	Armadillo-type fold	5
	4	IPR011989	Armadillo-like helical	5
	5	IPR011009	Protein kinase-like domain superfamily	4
	6	IPR000719	Protein kinase domain	4
	7	IPR012337	Ribonuclease H-like superfamily	4
	8	IPR005160	Ku70/Ku80 C-terminal arm	3
	9	IPR005161	Ku70/Ku80, N-terminal alpha/beta	3
	10	IPR006164	Ku70/Ku80 beta-barrel domain	3
Expanded in high-aggressive isolates	1	IPR027417	P-loop containing nucleoside triphosphate hydrolase	194
	2	IPR010285	DNA helicase Pif1-like	75
	3	IPR012337	Ribonuclease H-like superfamily	62
	4	IPR031825	RXLR phytopathogen effector protein	59
	5	IPR011009	Protein kinase-like domain superfamily	58
	6	IPR000719	Protein kinase domain	57
	7	IPR025476	Helitron helicase-like domain	45
	8	IPR008906	HAT, C-terminal dimerisation domain	41
	9	IPR014710	RmlC-like jelly roll fold	41
	10	IPR008271	Serine/threonine-protein kinase, active site	36

Table S8 Gene Ontology (GO) terms associated with the expanded and contracted gene families in the node containing the high- and low-aggressive isolates.

	Term_ID	Term_type	Term	FDR
Contracted proteins in low-aggressive isolates	GO:0008289	MF	lipid binding	1.20E-06
	GO:0003676		nucleic acid binding	0.0035
	GO:0016788		hydrolase activity, acting on ester bonds	0.021
Expanded proteins in low-aggressive isolates	GO:0000166	MF	nucleotide binding	0.00039
Contracted proteins in high-aggressive isolates	GO:0006259	BP	DNA metabolic process	0.015
	GO:0016817	MF	hydrolase activity, acting on acid anhydrides	0.018
	GO:0003677		DNA binding	0.018
	GO:0017111		nucleoside-triphosphatase activity	0.018
	GO:0016787		hydrolase activity	0.036
Expanded proteins in high-aggressive isolates	GO:0006259	BP	DNA metabolic process	1.30E-51
	GO:0006950		response to stress	1.80E-43
	GO:0050896		response to stimulus	7.80E-41
	GO:0042592		homeostatic process	4.90E-35
	GO:0065008		regulation of biological quality	2.40E-33
	GO:0006996		organelle organization	1.10E-32
	GO:0006139		nucleobase, nucleoside, nucleotide and nucleic acid metabolic process	4.40E-21
	GO:0044260		cellular macromolecule metabolic process	2.60E-20
	GO:0065007		biological regulation	1.20E-18
	GO:0043170		macromolecule metabolic process	5.70E-17
	GO:0006807		nitrogen compound metabolic process	9.00E-16
	GO:0044238		primary metabolic process	4.40E-11
	GO:0044237		cellular metabolic process	1.60E-10
	GO:0009987		cellular process	2.00E-08
	GO:0006464		protein modification process	1.90E-06
	GO:0008152		metabolic process	1.90E-06
	GO:0043412		macromolecule modification	1.50E-05
	GO:0044267		cellular protein metabolic process	0.0056
	GO:0006810		transport	0.026
	GO:0051179		localization	0.034
	GO:0006629		lipid metabolic process	0.038
	GO:0019538		protein metabolic process	0.038
	GO:0017111	MF	nucleoside-triphosphatase activity	6.20E-29
	GO:0016817		hydrolase activity, acting on acid anhydrides	1.90E-28
	GO:0016787		hydrolase activity	5.00E-12
	GO:0004672		protein kinase activity	6.30E-12
	GO:0000166		nucleotide binding	2.10E-08
	GO:0003677		DNA binding	7.90E-08
	GO:0016772		transferase activity, transferring phosphorus-containing groups	4.00E-06
	GO:0022857		transmembrane transporter activity	5.70E-06
	GO:0005215		transporter activity	1.80E-05
	GO:0005216		ion channel activity	0.00028
	GO:0022803		passive transmembrane transporter activity	0.00031
	GO:0003676		nucleic acid binding	0.00056
	GO:0003824		catalytic activity	0.0011
	GO:0016788		hydrolase activity, acting on ester bonds	0.022

Table S9 Summary of resequencing statistics.

	**MY-1**	**JHA1-7**	**KPC-7**	**PEP**	**Pc038**
Aggressiveness levels	Low	Medium	High	High	High
Sequencing depth	44	43	41	52	42
No. of filtered SNPs	1,028,593	1,008,744	1,010,595	1,017,990	1,051,265
No. of heterozygous SNPs	402,000	295,488	344,621	357,855	568,600
No. of filtered INDELs	247,406	239,270	241,527	241,392	255,907
No. of heterozygous INDELs	96,858	73,713	84,051	85,611	135,695
SNP density (SNPs per kb)	6.26	5.51	6.23	5.37	8.11
Nucleotide diversity (π)	0.004845	0.003744	0.004397	0.006825	0.003971
Transition / Transversion (Ts/Tv)	2.11	2.12	2.12	2.12	2.1
Synonymous variants	276,218	273,924	274,868	277,861	274,839
Missense variants	117,206	113,909	115,787	119,126	116,132
Nonsense variants	1,309	1,255	1,249	1,366	1,281
Variants in intergenic region	609,466	594,226	594,894	627,997	601,731

Table S10 Enirhced Gene Ontology (GO) terms for the genes containing more than 20 disruptive variants.

Term_ID	Term_type	Term	FDR
GO:0016887	MF	ATPase activity	4.90E-16
GO:0017111	MF	nucleoside-triphosphatase activity	3.20E-11
GO:0016818	MF	hydrolase activity, acting on acid anhydrides, in phosphorus-containing anhydrides	1.10E-10
GO:0016817	MF	hydrolase activity, acting on acid anhydrides	1.10E-10
GO:0016462	MF	pyrophosphatase activity	1.10E-10
GO:0005524	MF	ATP binding	6.10E-10
GO:0030554	MF	adenyl nucleotide binding	8.80E-10
GO:0032559	MF	adenyl ribonucleotide binding	9.80E-10
GO:0042623	MF	ATPase activity, coupled	2.10E-09
GO:0016820	MF	hydrolase activity, acting on acid anhydrides, catalyzing transmembrane movement of substances	6.00E-09
GO:0042626	MF	ATPase activity, coupled to transmembrane movement of substances	6.00E-09
GO:0043492	MF	ATPase activity, coupled to movement of substances	6.00E-09
GO:0035639	MF	purine ribonucleoside triphosphate binding	7.00E-09
GO:0017076	MF	purine nucleotide binding	8.60E-09
GO:0001883	MF	purine nucleoside binding	8.60E-09
GO:0001882	MF	nucleoside binding	8.60E-09
GO:0032549	MF	ribonucleoside binding	8.60E-09
GO:0032550	MF	purine ribonucleoside binding	8.60E-09
GO:0032555	MF	purine ribonucleotide binding	9.20E-09
GO:0015399	MF	primary active transmembrane transporter activity	1.10E-08
GO:0015405	MF	P-P-bond-hydrolysis-driven transmembrane transporter activity	1.10E-08
GO:0036094	MF	small molecule binding	8.80E-08
GO:0032553	MF	ribonucleotide binding	1.50E-07
GO:0097367	MF	carbohydrate derivative binding	1.50E-07
GO:0000166	MF	nucleotide binding	2.10E-07
GO:1901265	MF	nucleoside phosphate binding	2.10E-07
GO:0015267	MF	channel activity	3.90E-05
GO:0005216	MF	ion channel activity	3.90E-05
GO:0022838	MF	substrate-specific channel activity	3.90E-05
GO:0022803	MF	passive transmembrane transporter activity	3.90E-05
GO:0022857	MF	transmembrane transporter activity	8.90E-05
GO:0042625	MF	ATPase coupled ion transmembrane transporter activity	9.50E-05
GO:0022804	MF	active transmembrane transporter activity	9.50E-05
GO:0022853	MF	active ion transmembrane transporter activity	0.00023
GO:0016874	MF	ligase activity	0.00065
GO:0015662	MF	ATPase activity, coupled to transmembrane movement of ions, phosphorylative mechanism	0.00065
GO:0016787	MF	hydrolase activity	0.0012
GO:0003950	MF	NAD+ ADP-ribosyltransferase activity	0.0027
GO:0003678	MF	DNA helicase activity	0.0036
GO:0008094	MF	DNA-dependent ATPase activity	0.004
GO:0004003	MF	ATP-dependent DNA helicase activity	0.005
GO:0008570	MF	obsolete myosin ATPase activity	0.005
GO:0015079	MF	potassium ion transmembrane transporter activity	0.0058
GO:0005515	MF	protein binding	0.0058
GO:0005215	MF	transporter activity	0.0066
GO:0015075	MF	ion transmembrane transporter activity	0.0086
GO:0016757	MF	transferase activity, transferring glycosyl groups	0.01
GO:0070035	MF	purine NTP-dependent helicase activity	0.014
GO:0008026	MF	ATP-dependent helicase activity	0.014

GO:0022891	MF	substrate-specific transmembrane transporter activity	0.016
GO:0005261	MF	cation channel activity	0.019
GO:0005267	MF	potassium channel activity	0.02
GO:0004386	MF	helicase activity	0.022
GO:0043774	MF	coenzyme F420-2 alpha-glutamyl ligase activity	0.028
GO:0043773	MF	coenzyme F420-0 gamma-glutamyl ligase activity	0.028
GO:0008766	MF	UDP-N-acetylmuramoylalanyl-D-glutamyl-2,6-diaminopimelate-D-alanyl-D-alanine ligase activity	0.028
GO:0022836	MF	gated channel activity	0.028
GO:0018169	MF	ribosomal S6-glutamic acid ligase activity	0.028
GO:0070739	MF	protein-glutamic acid ligase activity	0.028
GO:0008186	MF	RNA-dependent ATPase activity	0.03
GO:0043142	MF	single-stranded DNA-dependent ATPase activity	0.03
GO:0017116	MF	single-stranded DNA-dependent ATP-dependent DNA helicase activity	0.03
GO:0004004	MF	ATP-dependent RNA helicase activity	0.03
GO:0042624	MF	ATPase activity, uncoupled	0.03
GO:0015462	MF	protein-transmembrane transporting ATPase activity	0.03
GO:0003910	MF	DNA ligase (ATP) activity	0.035
GO:0003909	MF	DNA ligase activity	0.035
GO:0043682	MF	copper-transporting ATPase activity	0.035
GO:0022892	MF	substrate-specific transporter activity	0.035
GO:0008047	MF	enzyme activator activity	0.035
GO:0004008	MF	copper-exporting ATPase activity	0.035
GO:0003774	MF	motor activity	0.035
GO:0005509	MF	calcium ion binding	0.041
GO:0003724	MF	RNA helicase activity	0.043
GO:0016881	MF	acid-amino acid ligase activity	0.048
GO:0030286	CC	dynein complex	0.0047
